# Novel Trifluoromethyl Pyrimidinone Compounds With Activity Against *Mycobacterium tuberculosis*

**DOI:** 10.3389/fchem.2021.613349

**Published:** 2021-04-29

**Authors:** Erik Hembre, Julie V. Early, Joshua Odingo, Catherine Shelton, Olena Anoshchenko, Junitta Guzman, Lindsay Flint, Devon Dennison, Matthew B. McNeil, Aaron Korkegian, Yulia Ovechkina, Paul Ornstein, Thierry Masquelin, Philip A. Hipskind, Tanya Parish

**Affiliations:** ^1^Lilly Research Laboratories, Eli Lilly and Company, Indianapolis, IN, United States; ^2^TB Discovery Research, Infectious Disease Research Institute, Seattle, WA, United States; ^3^Center for Global Infectious Disease Research, Seattle Children's Research Institute, Seattle, WA, United States; ^4^Apollo Drug Discovery Consulting, LLC, Northbrook, IL, United States

**Keywords:** tuberculosis, anti-tubercular, bactericidal ability, whole cell activity, pyrimidinones

## Abstract

The identification and development of new anti-tubercular agents are a priority research area. We identified the trifluoromethyl pyrimidinone series of compounds in a whole-cell screen against *Mycobacterium tuberculosis*. Fifteen primary hits had minimum inhibitory concentrations (MICs) with good potency IC_90_ is the concentration at which *M. tuberculosis* growth is inhibited by 90% (IC_90_ < 5 μM). We conducted a structure–activity relationship investigation for this series. We designed and synthesized an additional 44 molecules and tested all analogs for activity against *M. tuberculosis* and cytotoxicity against the HepG2 cell line. Substitution at the 5-position of the pyrimidinone with a wide range of groups, including branched and straight chain alkyl and benzyl groups, resulted in active molecules. Trifluoromethyl was the preferred group at the 6-position, but phenyl and benzyl groups were tolerated. The 2-pyridyl group was required for activity; substitution on the 5-position of the pyridyl ring was tolerated but not on the 6-position. Active molecules from the series demonstrated low selectivity, with cytotoxicity against eukaryotic cells being an issue. However, there were active and non-cytotoxic molecules; the most promising molecule had an MIC (IC_90_) of 4.9 μM with no cytotoxicity (IC_50_ > 100 μM). The series was inactive against Gram-negative bacteria but showed good activity against Gram-positive bacteria and yeast. A representative molecule from this series showed rapid concentration-dependent bactericidal activity against replicating *M. tuberculosis* bacilli with ~4 log kill in <7 days. Overall the biological properties were promising, if cytotoxicity could be reduced. There is scope for further medicinal chemistry optimization to improve the properties without major change in structural features.

## Introduction

Tuberculosis remains a major global health killer with >1.5 million deaths and 10 million new cases in 2018 (World Health Organization, 2020). The current drug treatment regimen involves multiple antibiotics over a lengthy period of at least 6 months. In addition, there are multidrug-resistant and extremely drug-resistant strains circulating, making the current drugs ineffective. Thus, there is an urgent need for new drugs active against the causative pathogen *Mycobacterium tuberculosis* (Gordon and Parish, 2018).

The identification and development of anti-tubercular agents has been an increasing priority in the research community; target-based and whole-cell screens have been developed, which enable the screening of large libraries to find novel scaffolds with desirable biological activities (Parish, 2020). Phenotypic screening in which compounds are tested directly against the virulent organism in order to find novel matter has been widely utilized in the last decade or so (Parish, 2020). From these screens, a large number of compounds series that inhibit the growth of *M. tuberculosis* have been identified and explored (Gold and Nathan, 2017; Grzelak et al., 2019; Parish, 2020). However, given the high attrition rate in drug discovery, the difficulty of killing *M. tuberculosis* (as opposed to inhibiting growth), and the fact that it exists in different physiological states during infection, there is still a need for additional chemical (Payne et al., 2007; Keiser and Purdy, 2017; Mandal et al., 2019).

We have previously developed high-throughput screening using fluorescent strains of *M. tuberculosis*, which enabled us to screen compound libraries (Ollinger et al., 2018). We identified several series with anti-tubercular activity; among these is the trifluoromethyl pyrimidinone series, which has potent activity *in vitro*. Pyrimidinones are an important class of heterocycles containing heteroatoms that are broadly useful in medicinal chemistry (Fruit and Besson, 2018). This scaffold contains a range of drug molecules in the disease areas of cancer (5-fluorouracil and tegafur) (Papanastasopoulos and Stebbing, 2014) and HIV such as raltegravir (MK0518) (Marinello et al., 2008). We report the synthesis and structure–activity relationship (SAR) of this novel heterocyclic scaffold.

## Materials and Methods

### General Chemistry Methods

Chemicals and solvents were purchased from commercial vendors. Analytical thin-layer chromatography (TLC) was performed with precoated TLC plates, air dried, and analyzed using a UV lamp (UV254/365 nm) and/or an aqueous solution of potassium permanganate for visualization. Flash chromatography was performed using a Combiflash Companion R_f_ (Teledyne ISCO) and prepacked silica gel columns. Mass-directed preparative high-performance liquid chromatography (HPLC) separations were performed using a Waters HPLC (2,545 binary gradient pumps, 515 HPLC makeup pump, 2,767 sample manager) connected to a Waters 2998 photodiode array and a Waters 3100 mass detector. Preparative HPLC separations were performed with a Gilson HPLC connected to a Gilson 155 UV/vis detector. HPLC chromatographic separations were conducted using Waters XBridge C18 columns 19 × 100 mm, 5 μm particle size, using 0.1% ammonia in water (solvent A) and acetonitrile (solvent B) as mobile phase. ^1^H NMR and ^13^C NMR spectra were recorded on a Bruker Advance II 500, 400, or 300 spectrometer operating at 500, 400, or 300 MHz (unless otherwise stated) using CDCl_3_ or dimethyl sulfoxide (DMSO)-*d*_6_ solutions. Chemical shifts (δ) are expressed in ppm recorded using the residual solvent as the internal reference in all cases. Signal splitting patterns described as singlet (s), doublet (d), triplet (t), multiplet (m), broad singlet (br, s), or a combination thereof. Coupling constants (J) quoted to the nearest 0.1 Hz. Low-resolution electrospray (ES) mass spectra were recorded on a Bruker Daltonics MicrOTOF mass spectrometer run in positive mode. High-resolution mass spectroscopy was performed using a Bruker Daltonics MicrOTOF mass spectrometer. Liquid chromatography–mass spectrometry (LC-MS) analysis and chromatographic separation were conducted with a Bruker Daltonics MicrOTOF mass spectrometer or an Agilent Technologies 1200 series HPLC connected to an Agilent Technologies 6130 quadrupole LC-MS where both instruments connected to an Agilent diode array detector. The columns used were Waters XBridge column (50 × 2.1 mm, 3.5 μm particle size), and the compounds were eluted with a gradient from 5 to 95% using acetonitrile in water with 0.1% ammonia. All final compounds had a purity of ≥95% as determined by the UV chromatogram (190–450 nm) obtained by LC-MS analysis. High-resolution ES measurements performed on a Bruker MicroT of mass spectrometer.

### Synthesis of 5-(4-Fluorobenzyl)-2-(Pyridin-2-yl)-6-(Trifluoromethyl)Pyrimidin-4(3H)-One (5, 0066241)

(i) Synthesis of ethyl 4,4,4-trifluoro-2-(4-fluorobenzyl)-3-oxobutanoate: 3-(4-Fluorophenyl)propanoyl chloride 86 (2.5 g, 13.4 mmol) was taken in dichloromethane (DCM) (20 mL) in a 250 mL round-bottom flask under N_2_. TFAA (2.8 mL, 20.1 mmol) and pyridine (2.1 mL, 26.8 mmol) were sequentially added to it. The reaction mixture was stirred at room temperature (RT) for 3 h. EtOH (4 mL) was added to the reaction mixture and kept stirring for another 12 h. The reaction mixture was concentrated under reduced pressure and triturated with hexane. This afforded ethyl 4,4,4-trifluoro-2-(4-fluorobenzyl)-3-oxobutanoate as a brown oil (2.25 g, crude) that was used as such for the next step without any further purification. MS: 291.0 (M-H)^+^. (ii) Et_3_N (5.4 mL, 37.5 mmol) was added to a solution of ethyl 4,4,4-trifluoro-2-(4-fluorobenzyl)-3-oxobutanoate (2.2 g, 7.5 mmol) and picolinimidamide hydrochloride **3** (1.18 g, 7.5 mmol) in EtOH (15 mL) at RT. The reaction mixture was then heated at 100°C and monitored by TLC analysis (50% EtOAc-hexane) until completion. The reaction mixture was then poured into ice water (20 g) and extracted with EtOAc (3 × 30 mL). The combined organic layer was dried over anhydrous Na_2_SO_4_ and concentrated under reduced pressure. The crude was purified by flash chromatography on silica gel (100–200 mesh) using 5% EtOAc-hexane as eluent to afford **5** as a white solid (500 mg, 19.0%). M.P. 183–185°C. ^1^H NMR (400 MHz, DMSO-d_6_): δ 13.01 (s, 1H), 8.77 (d, *J* = 4.0 Hz, 1H), 8.31 (d, *J* = 7.9 Hz, 1H), 8.06–8.10 (m, 1H), 7.67–7.70 (m, 1H), 7.23–7.26 (m, 2H), 7.07–7.12 (m, 2H), 3.98 (s, 2H); ^13^C NMR (100 MHz, DMSO-d_6_): δ 162.1, 160.8 (d, *J* = 248 Hz), 153.9, 149.3, 147.8, 146.9 (q, *J* = 32 Hz), 138.1, 134.3 (d, *J* = 2.8 Hz), 129.8 (d, *J* = 8.0 Hz), 127.0, 126.8, 122.7, 121.6 (q, *J* = 274 Hz), 115.0 (q, *J* = 21 Hz), 29.2. LCMS (ESI) m/z calculated for [C_17_H_11_F_4_N_3_O + H]^+^, 350.09; found 350.78.

### Synthesis of 2-(Pyridin-2-yl)-6-(Trifluoromethyl)Pyrimidin-4(3H)-One (16, 0496338)

Et_3_N (2.2 mL, 16.0 mmol) was added to a solution of ethyl 4,4,4-trifluoro-3-oxobutanoate (700 mg, 3.8 mmol) and picolinimidamide hydrochloride **3** (500 mg, 3.2 mmol) in EtOH (10 mL) at RT. The reaction mixture was then heated at 110°C and monitored by TLC analysis (EtOAc) until completion. The reaction mixture was evaporated to dryness and poured to ice cooled water (20 mL). It was extracted with DCM (3 × 20 mL). The combined organic layer was dried over anhydrous Na_2_SO_4_ and concentrated under reduced pressure. The crude was further purified by column chromatography on silica gel (60–120 mesh) using 25% EtOAc-hexane as eluent to afford **16** as a white solid (365 mg, 47%). M.P. 136–137°C ^1^H NMR (400 MHz, DMSO-d_6_): δ 12.91 (s, 1H), 8.78–8.79 (m, 1H), 8.31 (d, *J* = 7.9 Hz, 1H), 8.06–8.10 (m, 1H), 7.68–7.72 (m, 1H), 6.96 (s, 1H); ^13^C NMR (100 MHz, DMSO-d_6_): δ 160.9, 157.0, 151.1 (q, *J* = 35 Hz), 149.3, 147.6, 138.2, 127.3, 122.9, 120.7 (q, *J* = 273 Hz), 113.5. LCMS (ESI) m/z calculated for [C_10_H_6_F_3_N_3_O + H]^+^, 242.05; found 242.07.

### Synthesis of 2-(3-Hydroxypyridin-2-yl)-6-(Trifluoromethyl)Pyrimidin-4(3H)-One (17, 0504065)

(i) Synthesis of isoxazolo[5,4-b]pyridin-3-amine: Potassium tert-butoxide (2.7 g, 24.6 mmol) was taken in in anhydrous DMF (20 mL) in a 100-mL round-bottom flask under N_2_ and cooled it down to 0°C. 3-Fluoropicolinonitrile (2.0 g, 16.4 mmol) and N-hydroxyacetamide (1.7 g, 22.9 mmol) were sequentially added to it. The reaction mixture was stirred at 60°C and monitored by TLC analysis (50% EtOAc-hexane) until completion. Ice cold water (40 mL) was added to the reaction mixture and the reaction mixture was extracted with EtOAc (3 × 20 mL). The combined organic layer was dried over anhydrous Na_2_SO_4_ and concentrated under reduced pressure. The crude was purified by flash chromatography on silica gel (60–120 mesh) using 30% EtOAc-hexane as eluent to afford isoxazolo[5,4-b]pyridin-3-amine as a gray solid (1.5 g, 68%). ^1^H NMR (400 MHz, DMSO-d_6_): δ 8.80 (s, 1H), 7.92 (d, *J* = 7.9 Hz, 1H), 7.55–7.59 (m, 1H), 6.55 (bs, 2H). (ii) Synthesis of 3-hydroxypicolinimidamide: A par flask was charged with isoxazolo[5,4-b]pyridin-3-amine (1.5 g, 11.1 mmol) and MeOH (20 mL) followed by addition of Pd-C (20% wt/wt, 0.6 g). The flask was evacuated under vacuum and then purged with hydrogen. The reaction was stirred under hydrogen atmosphere (60 psi) for 24 h. The reaction was monitored by TLC. It was then filtered through sintered funnel with a pad of celite, washed with MeOH (30 mL) and concentrated under reduced pressure. It was recrystallized from diethyl ether/pentane to afford 3-hydroxypicolinimidamide as a pale brown solid (0.8 g, 65%). LC-MS: 137.99 (M+H)^+^; 78% (purity). (iii) Et_3_N (0.4 mL, 3.0 mmol) was added to a solution of ethyl 4,4,4-trifluoro-3-oxobutanoate **8** (322 mg, 1.7 mmol) and 3-hydroxypicolinimidamide (200 mg, 1.4 mmol) in EtOH (5 mL) at RT. The reaction mixture was then heated at 80°C and monitored by TLC analysis (50% EtOAc-hexane) until completion. The reaction mixture was evaporated to dryness. The crude was purified by flash chromatography on silica gel (60–120 mesh) using 30% EtOAc-hexane as eluent to afford **17** as an off-white solid (100 mg, 27%). M.P. 170–171°C. ^1^H NMR (400 MHz, DMSO-d_6_): δ 13.01 (bs, 1H), 12.12 (bs, 1H), 8.31 (d, *J* = 3.4 Hz, 1H), 7.60–7.63 (m, 1H), 7.55 (d, *J* = 8.4 Hz, 1H), 7.00 (s, 1H); ^13^C NMR (100 MHz, DMSO-d_6_): δ 160.1, 158.5, 156.9, 146.43 (q, *J* = 33 Hz), 140.8, 129.6, 129.4, 126.5, 120.4 (q, *J* = 274 Hz), 113.5. LCMS (ESI) m/z calculated for [C_10_H_6_F_3_N_3_O_2_ + H]^+^, 258.05; found 258.19.

### Synthesis of 6-(Trifluoromethyl)-[2,2′-Bipyrimidin]-4(3H)-One (18, 0504070)

(i) Synthesis of pyrimidine-2-carboximidamide: Pyrimidine-2-carbonitrile (500 mg, 4.7 mmol) was taken in anhydrous THF (10 mL) in a 50 mL round bottom flask under N_2_ and cooled it down to −20°C. Dropwise lithium bis(trimethylsilyl)amide (1.06 M in THF) (5.3 mL, 5.7 mmol) was added to it. The reaction mixture was stirred at RT for 18 h and monitored by TLC analysis (50% EtOAc-hexane). 6N HCl (10 mL) was added to the reaction mixture and kept it stirring at for 30 min. The reaction mixture was then extracted with EtOAc (2 × 20 mL). The combined organic layer was rejected and the aqueous layer was basified by NaOH solution to pH 13–14. The aqueous layer was extracted with EtOAc (2 × 30 mL). The combined organic layer dried over anhydrous Na_2_SO_4_ and concentrated under reduced pressure. It was further recrystallized from diethyl ether-pentane to afford pyrimidine-2-carboximidamide as a pale yellow solid (420 mg, 72%). ^1^H NMR (400 MHz, DMSO-d_6_): δ 8.95 (d, *J* = 4.8 Hz, 2H), 7.62–7.67 (m, 1H), 7.14 (bs, 3H). (ii) Et_3_N (0.5 mL, 3.2 mmol) was added to a solution of ethyl 4,4,4-trifluoro-3-oxobutanoate (353 mg, 1.9 mmol) and pyrimidine-2-carboximidamide (200 mg, 1.6 mmol) in EtOH (5 mL) at RT. The reaction mixture was then heated at 80°C and monitored by TLC analysis (80% EtOAc-hexane) until completion. The reaction mixture was evaporated to dryness. The crude was purified by flash chromatography on silica gel (60–120 mesh) using 50% EtOAc-hexane as eluent to afford **18** as a yellow solid (88 mg, 22%). M.P. 236–238°C. ^1^H NMR (400 MHz, DMSO-d_6_): δ 13.26 (s, 1H), 9.08 (d, *J* = 4.8 Hz, 2H), 7.76–7.79 (m, 1H), 7.06 (s, 1H); ^13^C NMR (100 MHz, DMSO-d_6_): δ 160.3, 158.2, 156.7, 156.3, 151.4 (q, *J* = 34 Hz), 123.4, 120.7 (q, *J* = 274 Hz), 114.5. LCMS (ESI) m/z calculated for [C_9_H_5_F_3_N_4_O + H]^+^, 243.17; found 243.8.

### Synthesis of 2-(3-Methoxypyridin-2-yl)-6-(Trifluoromethyl)Pyrimidin-4(3H)-One (19, 0499009)

**(**i) Synthesis of 3-methoxypicolinimidamide: 3-Methoxypicolinonitrile (1.0 g, 7.4 mmol) was taken in anhydrous THF (10 mL) in a 50-mL round-bottom flask under N_2_ and cooled it down to −20°C. Dropwise lithium bis(trimethylsilyl)amide (1.06 M in THF) (21 mL, 22.4 mmol) was added to it. The reaction mixture was stirred at RT and monitored by TLC analysis (50% EtOAc-hexane) until completion. 6N HCl (20 mL) was added to the reaction mixture and kept it stirring at for 30 min. The reaction mixture was then extracted with EtOAc (2 × 20 mL). The combined organic layer was rejected and the aqueous layer was basified by NaOH solution to pH 13–14. The aqueous layer was extracted with EtOAc (2 × 30 mL). The combined organic layer dried over anhydrous Na_2_SO_4_ and concentrated under reduced pressure. It was further recrystallized from ether-pentane to afford 3-methoxypicolinimidamide as a pale brown solid (700 mg, 62%). ^1^H NMR (400 MHz, DMSO-d_6_): δ 8.21 (bs, 1H), 7.63–7.65 (m, 1H), 7.42–7.53 (m, 2H), 7.03 (bs, 2H), 3.91 (s, 3H). (ii) Et_3_N (0.4 mL, 2.6 mmol mmol) was added to a solution of ethyl 4,4,4-trifluoro-3-oxobutanoate (280 mg, 1.5 mmol) and 3-methoxypicolinimidamide (200 mg, 1.3 mmol) in EtOH (5 mL) at RT. The reaction mixture was then heated at 80°C and monitored by TLC analysis (5% MeOH–DCM) until completion. The reaction mixture was evaporated to dryness. The crude was purified by flash chromatography on silica gel (60–120 mesh) using 2% MeOH–DCM as eluent to afford **19** as a white solid (80 mg, 22%). M.P. 178–179°C. ^1^H NMR (400 MHz, DMSO-d_6_): δ 13.31 (s, 1H), 8.28 (d, *J* = 4.4 Hz, 1H), 7.71 (d, *J* = 8.5 Hz, 1H), 7.61 (q, *J* = 8.5 Hz, 1H), 6.93 (s, 1H), 3.86 (s, 3H); ^13^C NMR (100 MHz, DMSO-d_6_): δ 161.2, 157.9, 154.2, 151.5 (q, *J* = 33 Hz), 140.9, 139.3, 127.2, 120.7 (q, *J* = 273 Hz), 120.6, 112.8, 56.1. LCMS (ESI) m/z calculated for [C_11_H_8_F_3_N_3_O_2_ + H]^+^, 272.06; found 272.0.

### Synthesis of 2-(Pyridin-3-yl)-6-(Trifluoromethyl)Pyrimidin-4(3H)-One (20, 0497740)

Et_3_N (0.5 mL, 3.2 mmol) was added to a solution of ethyl 4,4,4-trifluoro-3-oxobutanoate (250 mg, 1.6 mmol) and nicotinimidamide hydrochloride (294 mg, 1.6 mmol) in EtOH (5 mL) at RT. The reaction mixture was then heated at 80°C for 12 h and monitored by TLC analysis (50% EtOAc-hexane). The reaction mixture was evaporated to dryness. The crude was purified by column chromatography on silica gel (60–120 mesh) using 70% EtOAc-hexane as eluent to afford **20** as a white solid (100 mg, 26%). M.P. 276–277°C. ^1^H NMR (400 MHz, DMSO-d_6_): δ 13.52 (s, 1H), 9.23 (s, 1H), 8.78–8.79 (m, 1H), 8.44 (d, *J* = 8.2 Hz, 1H), 7.59–7.62 (m, 1H), 6.95 (s, 1H); ^13^C NMR (100 MHz, DMSO-d_6_): δ 164.1, 159.3, 153.1, 152.5 (q, *J* = 35 Hz), 149.4, 136.2, 128.6, 124.1, 121.1 (q, *J* = 273 Hz), 110.9. LCMS (ESI) m/z calculated for [C_10_H_6_F_3_N_3_O + H]^+^, 242.05; found 242.28.

### Synthesis of 2-(3-Chloropyridin-2-yl)-6-(Trifluoromethyl)Pyrimidin-4(3H)-One (21, 0498345)

(i) Synthesis of 3-chloropicolinimidamide: 3-Chloropicolinonitrile (500 mg, 3.6 mmol) was taken in anhydrous THF (20 mL) in a 50-mL round-bottom flask under N_2_ and cooled it down to −20°C. Dropwise lithium bis(trimethylsilyl)amide (1.06 M in THF) (10.2 mL, 10.8 mmol) was added to it. The reaction mixture was stirred at RT and monitored by TLC analysis (50% EtOAc-hexane) until completion. 6N HCl (10 mL) was added to the reaction mixture and kept it stirring at for 30 min. The reaction mixture was then extracted with EtOAc (2 × 20 mL). The combined organic layer was rejected and the aqueous layer was basified by NaOH solution to pH 13–14. The aqueous layer was extracted with EtOAc (2 × 30 mL). The combined organic layer dried over anhydrous Na_2_SO_4_ and concentrated under reduced pressure to afford 3-chloropicolinimidamide as a brown-colored solid (350 mg, 62%). ^1^H NMR (400 MHz, DMSO-d_6_): δ 8.50 (d, *J* = 4.6 Hz, 1H), 7.97 (d, *J* = 8.1 Hz, 1H), 7.45 (q, *J* = 4.6 Hz, 1H), 6.50 (bs, 3H). (ii) Et_3_N (0.3 mL, 1.9 mmol) was added to a solution of ethyl 4,4,4-trifluoro-3-oxobutanoate (210 mg, 1.1 mmol) and 3-chloropicolinimidamide (150 mg, 0.9 mmol) in EtOH (5 mL) at RT. The reaction mixture was then heated at 80°C and monitored by TLC analysis (70% EtOAc-hexane) until completion. The reaction mixture was evaporated to dryness. The crude was purified by flash chromatography on silica gel (60–120 mesh) using 30% EtOAc-hexane as eluent to afford **21** as an off-white solid (80 mg, 30%). M.P. 163–164°C. ^1^H NMR (400 MHz, DMSO-d_6_): δ 13.62 (s, 1H), 8.69 (q, *J* = 4.6 Hz, 1H), 8.19 (q, *J* = 8.2 Hz, 1H), 7.67–7.70 (m, 1H), 7.04 (s, 1H); ^13^C NMR (100 MHz, DMSO-d_6_): δ 161.5, 157.6, 151.1 (q, *J* = 43 Hz), 147.8, 138.9, 129.8, 127.3, 120.6 (q, *J* = 274 Hz), 113.6. LCMS (ESI) m/z calculated for [C_10_H_5_ClF_3_N_3_O + H]^+^, 276.02; found 276.23.

### Synthesis of 5-Methyl-2-(Pyridin-3-yl)-6-(Trifluoromethyl)Pyrimidin-4(3H)-One (22, 0497401)

Et_3_N (0.9 mL, 6.5 mmol) was added to a solution of ethyl 4,4,4-trifluoro-2-methyl-3-oxobutanoate (250 mg, 1.3 mmol) and nicotinimidamide hydrochloride (198 mg, 1.3 mmol) in EtOH (10 mL) at RT. The reaction mixture was then heated at 110°C for 5 h and monitored by TLC analysis (5% MeOH–DCM) until completion. The reaction mixture was evaporated to dryness and poured to ice cooled water (20 mL). It was extracted with DCM (3 × 20 mL). The combined organic layer was dried over anhydrous Na_2_SO_4_ and concentrated under reduced pressure. The crude was further purified by column chromatography on silica gel (60–120 mesh) using 20% EtOAc-hexane as eluent to afford **19** as a white solid (150 mg, 58%). The crude was purified by flash chromatography on silica gel (60–120 mesh) using 19% EtOAc-hexane as eluent to afford **23** as a white solid (140 mg, 43%). M.P. 213–215°C. ^1^H NMR (400 MHz, DMSO-d_6_): δ 13.48 (s, 1H), 9.22 (d, *J* = 1.4 Hz, 1H), 8.74–8.76 (m, 1H), 8.42 (d, *J* = 8.0 Hz, 1H), 7.56–7.60 (m, 1H), 2.16 (d, *J* = 2.2 Hz, 3H); ^13^C NMR (100 MHz, DMSO-d_6_): δ 164.1, 153.9, 152.2, 148.7, 146.8 (q, *J* = 24.8 Hz), 135.4, 128.0, 123.9 (q, *J* = 275 Hz), 123.6, 122.6, 10.1. LCMS (ESI) m/z calculated for [C_11_H_8_F_3_N_3_O + H]^+^, 256.07; found 256.28.

### Synthesis of 5-Methyl-2-(Pyridin-4-yl)-6-(Trifluoromethyl)Pyrimidin-4(3H)-One (23, 0497305)

Na_2_CO_3_ (210 mg, 2.0 mmol mmol) was added to a solution of ethyl 4,4,4-trifluoro-2-methyl-3-oxobutanoate (400 mg, 2.0 mmol) and isonicotinimidamide hydrochloride (310 mg, 2.0 mmol) in EtOH (4 mL) at RT. The reaction mixture was then heated at 80°C for 18 h and monitored by TLC analysis (50% EtOAc-hexane) until completion. The reaction mixture was evaporated to dryness. The crude was purified by column chromatography on silica gel (60–120 mesh) using 25% EtOAc-hexane as eluent to afford **23** as a white solid (200 mg, 39%). M.P. 228–230°C. ^1^H NMR (400 MHz, DMSO-d_6_): δ 13.53 (s, 1H), 8.79 (d, *J* = 5.4 Hz, 2H), 8.03 (d, *J* = 5.4 Hz, 2H), 2.18 (d, *J* = 1.4 Hz, 3H); ^13^C NMR (100 MHz, DMSO-d_6_): δ 164.3, 153.9, 150.4, 146.9 (q, *J* = 32 Hz), 139.2, 123.4, 121.3, 121.7 (q, *J* = 274.6 Hz), 10.2 (q, *J* = 3.0 Hz). LCMS (ESI) m/z calculated for [C_11_H_8_F_3_N_3_O + H]^+^, 256.07; found 256.46.

### Synthesis of 2-Phenyl-6-(Trifluoromethyl)Pyrimidin-4(3H)-One (24, 0143990)

Et_3_N (0.1 mL, 0.9 mmol) was added to a solution of ethyl 4,4,4-trifluoro-3-oxobutanoate (211 mg, 1.1 mmol) and benzimidamide hydrochloride (150 mg, 0.9 mmol) in EtOH (4 mL) at RT. The reaction mixture was then heated at 80°C and monitored by TLC analysis (50% EtOAc-hexane) until completion. The reaction mixture was evaporated to dryness. The crude was purified by flash chromatography on silica gel (60–120 mesh) using 15% EtOAc-hexane as eluent to afford **24** as a white solid (90 mg, 39%). M.P. 225–227°C. ^1^H NMR (400 MHz, DMSO-d_6_): δ 13.32 (s, 1H), 8.13 (d, *J* = 6.9 Hz, 2H), 7.62–7.66 (m, 1H), 7.55–7.58 (m, 2H), 6.87 (s, 1H); ^13^C NMR (100 MHz, DMSO-d_6_): δ 162.4, 159.7, 151.8, 132.4, 131.4, 128.8, 128.1, 120.7 (q, *J* = 273 Hz), 111.2. LCMS (ESI) m/z calculated for [C_11_H_7_F_3_N_2_O + H]^+^, 241.06; found 241.34.

### Synthesis of 2-(3-Fluoropyridin-2-yl)-6-(Trifluoromethyl)Pyrimidin-4(3H)-One (26, 0499232)

**(**i) Synthesis of 3-fluoropicolinimidamide: 3-Fluoropicolinonitrile (2.0 g, 16.4 mmol) was taken in anhydrous THF (20 mL) in a 100-mL round-bottom flask under N_2_ and cooled it down to −20°C. Dropwise lithium bis(trimethylsilyl)amide (1.06 M in THF) (30.9 mL, 32.8 mmol) was added to it. The reaction mixture was stirred at RT and monitored by TLC analysis (50% EtOAc-hexane) until completion. 6N HCl (30 mL) was added to the reaction mixture and kept it stirring at for 30 min. The reaction mixture was then extracted with EtOAc (2 × 20 mL). The combined organic layer was rejected and the aqueous layer was basified by NaOH solution to pH 13–14. The aqueous layer was extracted with EtOAc (2 × 30 mL). The combined organic layer dried over anhydrous Na_2_SO_4_ and concentrated under reduced pressure. It was further recrystallized from ether-pentane to afford 3-fluoropicolinimidamide as a brown solid (900 mg, 40%). MS: 139.91 (M+H)^+^. (ii) Et_3_N (3.2 mL, 22.8 mmol) was added to a solution of ethyl 4,4,4-trifluoro-3-oxobutanoate (1.0 g, 5.7 mmol) and 3-fluoropicolinimidamide (800 mg, 5.7 mmol) in EtOH (20 mL) at RT. The reaction mixture was then heated at 80°C and monitored by TLC analysis (50% EtOAc-hexane) until completion. The reaction mixture was evaporated to dryness. The crude was purified by flash chromatography on silica gel (60–120 mesh) using 24% EtOAc-hexane as eluent to afford **30** as a white solid (150 mg, 10%). M.P. 178–179°C. ^1^H NMR (400 MHz, DMSO-d_6_): δ 13.29 (s, 1H), 8.62 (d, *J* = 4.3 Hz, 1H), 8.06 (t, *J* = 9.5 Hz, 1H), 7.76–7.80 (m, 1H), 7.01 (s, 1H); ^13^C NMR (100 MHz, DMSO-d_6_): δ 161.2, 157.9 (d, *J* = 266 Hz), 155.5, 150.6 (q, *J* = 35 Hz), 145.5 (d, *J* = 5 Hz), 137.1, 128.9 (d, *J* = 4.6 Hz), 126.1 (d, *J* = 19 Hz), 120.6 (q, *J* = 273 Hz), 113.5. LCMS (ESI) m/z calculated for [C_10_H_5_F_4_N_3_O + H]^+^, 260.04; found 260.11.

### Synthesis of 2-Cyclohexyl-6-(Trifluoromethyl)Pyrimidin-4(3H)-One (27, 0504071)

(i) Synthesis of N′-hydroxycyclohexanecarboximidamide (116): Hydroxylamine (1.9 g, 27.5 mmol) was taken in EtOH (20 mL) in a 100-mL round-bottom flask under N_2_. Cyclohexanecarbonitrile (2.0 g, 18.3 mmol) and Et_3_N (3.3 mL, 22.9 mmol) were sequentially added to it. The reaction mixture was refluxed and monitored by TLC analysis (50% EtOAc-hexane) until completion. The reaction mixture was concentrated under reduced pressure and the crude was purified by flash chromatography on silica gel (60–120 mesh) using 20% EtOAc-hexane as eluent to afford N′-hydroxycyclohexanecarboximidamide as a gray solid (820 mg, 31%). ^1^H NMR (400 MHz, DMSO-d_6_): δ 8.67 (s, 1H), 5.21 (bs, 2H), 1.92–1.98 (m, 1H), 1.62–1.80 (m, 4H), 1.50–1.59 (m, 1H), 1.35–1.38 (m, 2H), 1.13–1.24 (m, 3H). (ii) Synthesis of cyclohexanecarboximidamide: A par flask was charged with N′-hydroxycyclohexanecarboximidamide (1.5 g, 11.1 mmol) and MeOH (20 mL) followed by addition of Pd-C (20% wt/wt, 0.6 g). The flask was evacuated under vacuum and then purged with hydrogen. The reaction was stirred under hydrogen atmosphere (60 psi) for 24 h. The reaction was monitored by TLC. It was then filtered through sintered funnel with a pad of celite, washed with MeOH (30 mL) and concentrated under reduced pressure. It was recrystallized from diethyl ether/pentane to afford cyclohexanecarboximidamide as a pale brown solid (0.56 g, 77%). MS m/z 127.2 [M^−^+ H]. (iii) Et_3_N (0.4 mL, 3.0 mmol) was added to a solution of ethyl 4,4,4-trifluoro-3-oxobutanoate (337 mg, 1.7 mmol) and cyclohexanecarboximidamide (200 mg, 1.4 mmol) in EtOH (5 mL) at RT. The reaction mixture was then heated at 80°C and monitored by TLC analysis (50% EtOAc-hexane) until completion. The reaction mixture was evaporated to dryness. The crude was purified by flash chromatography on silica gel (60–120 mesh) using 30% EtOAc-hexane as eluent to afford **27** as a white solid (150 mg, 38%). M.P. 198–199°C. ^1^H NMR (400 MHz, DMSO-d_6_): δ 12.92 (s, 1H), 6.68 (s, 1H), 2.56–2.62 (m, 1H), 1.82–1.85 (m, 2H), 1.75–1.78 (m, 2H), 1.64–1.66 (m, 1H), 1.44–1.53 (m, 2H), 1.18–1.31 (m, 3H); ^13^C NMR (100 MHz, DMSO-d_6_): δ 168.9, 162.2, 151.9 (d, *J* = 35 Hz), 121.2 (q, *J* = 273 Hz), 111.4, 43.0, 30.4, 25.7, 25.5. LCMS (ESI) m/z calculated for [C_11_H_13_F_3_N_2_O–H]^+^, 245.09; found 245.01.

### Synthesis of 2-(2-Fluorophenyl)-6-(Trifluoromethyl)Pyrimidin-4(3H)-One (28, 0497802)

Et_3_N (0.3 mL, 2.5 mmol) was added to a solution of ethyl 4,4,4-trifluoro-3-oxobutanoate (150 mg, 0.8 mmol) and 2-fluorobenzimidamide hydrochloride (190 mg, 1.0 mmol) in EtOH (5 mL) at RT. The reaction mixture was then heated at 110°C for 12 h and monitored by TLC analysis (30% EtOAc-hexane). The reaction mixture was evaporated to dryness and poured to ice cooled water (20 mL). It was extracted with DCM (3 × 20 mL). The combined organic layer was dried over anhydrous Na_2_SO_4_ and concentrated under reduced pressure. The crude was purified by flash chromatography on silica gel (60–120 mesh) using 18% EtOAc-hexane as eluent to afford **28** as a white solid (65 mg, 31%). M.P. 168–169°C. ^1^H NMR (400 MHz, DMSO-d_6_): δ 13.42 (s, 1H), 7.75 (t, *J* = 7.3 Hz, 1H), 7.64–7.69 (m, 1H), 7.36–7.43 (m, 2H), 6.92 (s, 1H); ^13^C NMR (100 MHz, DMSO-d_6_): δ 161.8, 160.7, 158.2, 157.0, 151.5 (q, *J* = 35 Hz), 133.8 (d, *J* = 8.5 Hz), 131.1, 124.7 (d, *J* = 3.3 Hz), 120.9, 117.7 (q, *J* = 273 Hz), 116.3 (d, *J* = 21 Hz). LCMS (ESI) m/z calculated for [C_11_H_6_F_4_N_2_O + H]^+^, 259.05; found 259.34.

### Synthesis of 2-(2,6-Dimethylphenyl)-6-(Trifluoromethyl)Pyrimidin-4(3H)-One (29, 0504068)

(i) Synthesis of N-hydroxy-2,6-dimethylbenzimidamide: Hydroxylamine (1.59 g, 22.9 mmol) was taken in EtOH (20 mL) in a 100-mL round-bottom flask under N_2_. 2,6-Dimethylbenzonitrile (2.0 g, 15.2 mmol) and Et_3_N (3.3 mL, 22.9 mmol) were sequentially added to it. The reaction mixture was refluxed and monitored by TLC analysis (50% EtOAc-hexane) until completion. The reaction mixture was concentrated under reduced pressure and the crude was purified by flash chromatography on silica gel (60–120 mesh) using 20% EtOAc-hexane as eluent to afford N-hydroxy-2,6-dimethylbenzimidamide as a gray solid (600 mg, 24%). LC-MS: 165.07 (M+H^+^); 98.79% (purity). (ii) Synthesis of 2,6-dimethylbenzimidamide: A par flask was charged with N-hydroxy-2,6-dimethylbenzimidamide (600 mg, 3.6 mmol) and MeOH (20 mL) followed by addition of Pd-C (20% wt/wt, 300 mg). The flask was evacuated under vacuum and then purged with hydrogen. The reaction was stirred under hydrogen atmosphere (60 psi) at RT for 48 h. The reaction was monitored by TLC. It was then filtered through sintered funnel with a pad of celite, washed with MeOH (30 mL) and concentrated under reduced pressure to afford Synthesis of 2,6-dimethylbenzimidamide: as an off-white solid (550 mg, crude). This was then used in the next step without further purification. ^1^H NMR (400 MHz, DMSO-d_6_): δ 7.83 (s, 1H), 7.46 (s, 1H), 7.11–7.15 (m, 2H), 7.01–7.03 (m, 2H), 2.24 (s, 6H). Et_3_N (0.5 mL, 3.4 mmol) was added to a solution of ethyl 4,4,4-trifluoro-3-oxobutanoate (375 mg, 2.0 mmol) and 2,6-dimethylbenzimidamide (250 mg, 1.7 mmol) in EtOH (5 mL) at RT. The reaction mixture was then heated at 80°C for 18 h and monitored by TLC analysis (50% EtOAc-hexane). The reaction mixture was evaporated to dryness. The crude was purified by flash chromatography on silica gel (60–120 mesh) using 20% EtOAc-hexane as eluent to afford **29** as a white solid (44 mg, 10%). M.P. 201–202°C. ^1^H NMR (400 MHz, DMSO-d_6_): δ ^1^H NMR (400 MHz, DMSO-d_6_): δ 13.35 (s, 1H), 7.33 (t. *J* = 7.5 Hz, 1H), 7.17 (d. *J* = 7.6 Hz, 2H), 6.89 (s, 1H), 2.14 (s, 6H); ^13^C NMR (100 MHz, DMSO-d_6_): δ 161.6, 161.0, 151.3 (q, *J* = 32 Hz), 135.4, 133.0, 129.8, 127.5, 120.7 (q, *J* = 274 Hz), 112.1, 18.9. LCMS (ESI) m/z calculated for [C_13_H_12_F_3_N_2_O + H]^+^, 269.09; found 269.25.

### Synthesis of 2-(o-Tolyl)-6-(Trifluoromethyl)Pyrimidin-4(3H)-One (30, 0499224)

(i) Synthesis of N-hydroxy-2-methylbenzimidamide: Hydroxylamine (2.37 g, 34.2 mmol) was taken in EtOH (20 mL) in a 100-mL round-bottom flask under N_2_. 2-Methylbenzonitrile (2.0 g, 17.1 mmol) and sodium carbonate (2.2 g, 20.5 mmol) were sequentially added to it. The reaction mixture was refluxed and monitored by TLC analysis (50% EtOAc-hexane) until completion. The reaction mixture was concentrated under reduced pressure and the crude was purified by flash chromatography on silica gel (60–120 mesh) using 20% EtOAc-hexane as eluent to afford N-hydroxy-2-methylbenzimidamide as a gray solid (800 mg, 31%). MS: 151.13 (M+H)^+^. (ii) Synthesis of 2-methylbenzimidamide: A par flask was charged with N-hydroxy-2-methylbenzimidamide (800 mg, 5.3 mmol) and MeOH (20 mL) followed by addition of Pd-C (10% wt/wt, 200 mg). The flask was evacuated under vacuum and then purged with hydrogen. The reaction was stirred under hydrogen atmosphere (60 psi) at RT for 8 h. The reaction was monitored by TLC. It was then filtered through sintered funnel with a pad of celite, washed with MeOH (30 mL) and concentrated under reduced pressure to afford 2-methylbenzimidamide: as a brown solid (280 mg, crude). This was then used in the next step without further purification. ^1^H NMR (400 MHz, DMSO-d_6_): δ 7.68 (bs, 1H), 7.28–7.38 (m, 4H), 7.18–7.26 (m, 2H), 2.36 (s, 3H). (iii) Synthesis of N-hydroxy-2-methylbenzimidamide: Hydroxylamine (2.37 g, 34.2 mmol) was taken in EtOH (20 mL) in a 100-mL round-bottom flask under N_2_. 2-Methylbenzonitrile (2.0 g, 17.1 mmol) and sodium carbonate (2.2 g, 20.5 mmol) were sequentially added to it. The reaction mixture was refluxed and monitored by TLC analysis (50% EtOAc-hexane) until completion. The reaction mixture was concentrated under reduced pressure and the crude was purified by flash chromatography on silica gel (60–120 mesh) using 20% EtOAc-hexane as eluent to afford N-hydroxy-2-methylbenzimidamide as a gray solid (800 mg, 31%). MS: 151.13 (M+H)^+^. (ii) Synthesis of 2-methylbenzimidamide: A par flask was charged with N-hydroxy-2-methylbenzimidamide (800 mg, 5.3 mmol) and MeOH (20 mL) followed by addition of Pd-C (10% wt/wt, 200 mg). The flask was evacuated under vacuum and then purged with hydrogen. The reaction was stirred under hydrogen atmosphere (60 psi) at RT for 8 h. The reaction was monitored by TLC. It was then filtered through sintered funnel with a pad of celite, washed with MeOH (30 mL) and concentrated under reduced pressure to afford 2-methylbenzimidamide: as a brown solid (280 mg, crude). This was then used in the next step without further purification. ^1^H NMR (400 MHz, DMSO-d_6_): δ 7.68 (bs, 1H), 7.28–7.38 (m, 4H), 7.18–7.26 (m, 2H), 2.36 (s, 3H). Et_3_N (0.4 mL, 2.6 mmol) was added to a solution of ethyl 4,4,4-trifluoro-3-oxobutanoate (329 mg, 1.8 mmol) and 2-methylbenzimidamide (200 mg, 1.5 mmol) in EtOH (5 mL) at RT. The reaction mixture was then heated at 80°C and monitored by TLC analysis (50% EtOAc-hexane) until completion. The reaction mixture was evaporated to dryness. The crude was purified by flash chromatography on silica gel (60–120 mesh) using 25% EtOAc-hexane as eluent to afford **30** as a white solid (70 mg, 18%). M.P. 155–156°C. ^1^H NMR (400 MHz, DMSO-d_6_): δ 13.28 (s, 1H), 7.45–7.51 (m, 2H), 7.32–7.37 (m, 2H), 6.86 (s, 1H), 2.37 (s, 3H); ^13^C NMR (100 MHz, DMSO-d_6_): δ 161.8, 151.2 (q, *J* = 34 Hz), 148.3, 136.4, 132.5, 130.8, 130.7, 129.4, 125.8, 120.7 (q, *J* = 273 Hz), 111.5, 19.4. LCMS (ESI) m/z calculated for [C_12_H_9_F_3_N_2_O + H]^+^, 255.07; found 255.25.

### Synthesis of 2-(2-Hydroxyphenyl)-6-(Trifluoromethyl)Pyrimidin-4(3H)-One (31, 0498338)

(i) Synthesis of benzo[d]isoxazol-3-amine: Potassium tert-butoxide (1.38 g, 12.3 mmol) was taken in in anhydrous DMF (20 mL) in a 100-mL round-bottom flask under N_2_ and cooled it down to 0°C. 2-Fluorobenzonitrile (1.0 g, 8.2 mmol) and N-hydroxyacetamide (867 mg, 11.5 mmol) were sequentially added to it. The reaction mixture was stirred at 50°C and monitored by TLC analysis (50% EtOAc-hexane) until completion. Ice cold water (40 mL) was added to the reaction mixture and the reaction mixture was extracted with EtOAc (3 × 20 mL). The combined organic layer was dried over anhydrous Na_2_SO_4_ and concentrated under reduced pressure to afford benzo[d]isoxazol-3-amine as a brown-colored solid (900 mg, crude). ^1^H NMR (400 MHz, DMSO-d_6_): δ 7.82 (d, *J* = 7.9 Hz, 1H), 7.54–7.56 (m, 1H), 7.43–7.45 (m, 1H), 7.24 (t, *J* = 7.2 Hz, 1H), 6.40 (bs, 2H). (ii) Synthesis of 2-hydroxybenzimidamide: A par flask was charged with benzo[d]isoxazol-3-amine 55 (0.15 g, 0.73 mmol) and MeOH (10 mL) followed by addition of Pd-C (10% wt/wt, 0.1 g). The flask was evacuated under vacuum and then purged with hydrogen. The reaction was stirred under hydrogen atmosphere (30 psi) for 18 h. The reaction was monitored by TLC. It was then filtered through sintered funnel with a pad of celite, washed with MeOH (30 mL) and concentrated under reduced pressure. It was recrystallized from diethyl ether/pentane to afford 2-hydroxybenzimidamide as a pale brown solid (0.6 g, 65%). MS: 136.93 (M+H)^+^. (iii) Et_3_N (0.4 mL, 3.0 mmol) was added to a solution of ethyl 4,4,4-trifluoro-3-oxobutanoate (324 mg, 1.8 mmol) and 2-hydroxybenzimidamide (200 mg, 1.5 mmol) in EtOH (5 mL) at RT. The reaction mixture was then heated at 80°C and monitored by TLC analysis (50% EtOAc-hexane) until completion. The reaction mixture was evaporated to dryness. The crude was purified by flash chromatography on silica gel (60–120 mesh) using 40% EtOAc-hexane as eluent to afford **31** as an off-white solid (100 mg, 27%). M.P. 236–238°C. ^1^H NMR (400 MHz, DMSO-d_6_): δ 12.39 (s, 1H), 8.06 (d, *J* = 7.6 Hz, 1H), 7.47 (t, *J* = 7.5 Hz, 1H), 6.97–7.03 (m, 2H), 6.88 (s, 1H); ^13^C NMR (100 MHz, DMSO-d_6_): δ 162.5, 160.0, 158.2, 151.2 (q, *J* = 42 Hz), 134.3, 129.3, 120.6 (q, *J* = 273 Hz), 119.5, 117.5, 115.8, 109.7. LCMS (ESI) m/z calculated for [C_11_H_7_F_3_N_3_O_2_ + H]^+^, 257.05; found 257.26.

### Synthesis of 5-Methyl-6-(Trifluoromethyl)Pyrimidin-4(3H)-One (32, 0496404)

Et_3_N (1.8 mL, 13.0 mmol) was added to a solution of ethyl 4,4,4-trifluoro-2-methyl-3-oxobutanoate (250 mg, 1.3 mmol) and formimidamide hydrochloride (101 mg, 1.3 mmol) in EtOH (3 mL) at RT. The reaction mixture was then heated at 80°C and monitored by TLC analysis (50% EtOAc-hexane) until completion. The reaction mixture was evaporated to dryness. The crude was purified by column chromatography on silica gel (60–120 mesh) using 25% EtOAc-hexane as eluent to afford **32** as a white solid (190 mg, 84%). M.P. 183–185°C. ^1^H NMR (400 MHz, DMSO-d_6_): δ 13.05 (s, 1H), 8.22 (s, 1H), 2.08 (s, 3H); ^13^C NMR (100 MHz, DMSO-d_6_): δ 161.9, 147.9, 146.3 (q, *J* = 32 Hz), 125.3, 121.8 (q, *J* = 275 Hz), 10.1 (q, *J* = 3 Hz). LCMS (ESI) m/z calculated for [C_6_H_5_F_3_N_2_O + H]^+^, 179.12; found 179.35.

### Synthesis of 2-(3-Hydroxypyridin-2-yl)-5-Methyl-6-(Trifluoromethyl)Pyrimidin-4(3H)-One (33, 0504069)

(i) Synthesis of Isoxazolo[5,4-b]Pyridin-3-Amine: Potassium tert-butoxide (2.7 g, 24.6 mmol) was taken in in anhydrous DMF (20 mL) in a 100-mL round-bottom flask under N_2_ and cooled it down to 0°C. 3-Fluoropicolinonitrile (2.0 g, 16.4 mmol) and N-hydroxyacetamide (1.7 g, 22.9 mmol) were sequentially added to it. The reaction mixture was stirred at 60°C and monitored by TLC analysis (50% EtOAc-hexane) until completion. Ice cold water (40 mL) was added to the reaction mixture and the reaction mixture was extracted with EtOAc (3 × 20 mL). The combined organic layer was dried over anhydrous Na_2_SO_4_ and concentrated under reduced pressure. The crude was purified by flash chromatography on silica gel (60–120 mesh) using 30% EtOAc-hexane as eluent to afford isoxazolo[5,4-b]pyridin-3-amine as a gray solid (1.5 g, 68%). ^1^H NMR (400 MHz, DMSO-d_6_): δ 8.80 (s, 1H), 7.92 (d, *J* = 7.9 Hz, 1H), 7.55–7.59 (m, 1H), 6.55 (bs, 2H). (ii) Synthesis of 3-hydroxypicolinimidamide: A par flask was charged with isoxazolo[5,4-b]pyridin-3-amine (1.5 g, 11.1 mmol) and MeOH (20 mL) followed by addition of Pd-C (20% wt/wt, 0.6 g). The flask was evacuated under vacuum and then purged with hydrogen. The reaction was stirred under hydrogen atmosphere (60 psi) for 24 h. The reaction was monitored by TLC. It was then filtered through sintered funnel with a pad of celite, washed with MeOH (30 mL) and concentrated under reduced pressure. It was recrystallized from diethyl ether/pentane to afford 3-hydroxypicolinimidamide as a pale brown solid (0.8 g, 65%). LC-MS: 137.99 (M+H)^+^; 78% (purity). (iii) Et_3_N (0.4 mL, 3.0 mmol) was added to a solution of ethyl 4,4,4-trifluoro-2-methyl-3-oxobutanoate (337 mg, 1.7 mmol) and 3-hydroxypicolinimidamide (200 mg, 1.4 mmol) in EtOH (5 mL) at RT. The reaction mixture was then heated at 80°C and monitored by TLC analysis (50% EtOAc-hexane) until completion. The reaction mixture was evaporated to dryness. The crude was purified by flash chromatography on silica gel (60–120 mesh) using 30% EtOAc-hexane as eluent to afford **33** as a white solid (150 mg, 38%). M.P. 176–178°C. ^1^H NMR (400 MHz, DMSO-d_6_): δ 12.97 (bs, 1H), 12.36 (bs, 1H), 8.29 (d, *J* = 4.0 Hz, 1H), 7.57–7.60 (m, 1H), 7.52 (d, *J* = 8.4 Hz, 1H), 2.16 (d, *J* = 2.1 Hz, 3H)); ^13^C NMR (100 MHz, DMSO-d_6_): δ 161.4, 156.7, 154.7, 142.9 (q, *J* = 35 Hz), 140.7, 129.5, 129.1, 126.2, 125.2, 121.4 (q, *J* = 274 Hz), 10.4 (d, *J* = 2.0 Hz). LCMS (ESI) m/z calculated for [C_11_H_9_F_3_N_3_O_2_ + H]^+^, 272.06; found 272.18.

### Synthesis of 2-(2-Methoxyphenyl)-5-Methyl-6-(Trifluoromethyl)Pyrimidin-4(3H)-One (34, 0497565)

(i) Synthesis of 2-methoxybenzimidamide: 2-Methoxybenzonitrile (1.0 g, 7.5 mmol) was taken in anhydrous THF (20 mL) in a 50-mL round-bottom flask under N_2_ and cooled it down to −20°C. Dropwise lithium bis(trimethylsilyl)amide (1.06 M in THF) (8.5 mL, 9.0 mmol) was added to it. The reaction mixture was stirred at RT and monitored by TLC analysis (50% EtOAc-hexane) until completion. 6N HCl (10 mL) was added to the reaction mixture and kept it stirring at for 30 min. The reaction mixture was then extracted with EtOAc (2 × 20 mL). The combined organic layer was rejected and the aqueous layer was basified by NaOH solution to pH 13–14. The aqueous layer was extracted with EtOAc (2 × 30 mL). The combined organic layer dried over anhydrous Na_2_SO_4_ and concentrated under reduced pressure to afford 2-methoxybenzimidamide as an off-white solid (350 mg, 31%). ^1^H NMR (400 MHz, DMSO-d_6_): δ 7.45 (d, *J* = 7.5 Hz, 1H), 7.33–7.38 (m, 1H), 7.06 (d, *J* = 8.3 Hz, 1H), 6.95 (t, *J* = 7.4 Hz, 1H), 6.36 (bs, 3H), 3.80 (s, 3H). LCMS (ESI) m/z calculated for [C_8_H_10_N_2_O + H]^+^, 151.09; found 151.07. (ii) Et_3_N (0.1 mL, 0.8 mmol) was added to a solution of ethyl 4,4,4-trifluoro-2-methyl-3-oxobutanoate (158 mg, 0.8 mmol) and 2-methoxybenzimidamide (120 mg, 0.8 mmol) in EtOH (5 mL) at RT. The reaction mixture was then heated at 80°C for 18 h and monitored by TLC analysis (80% EtOAc-hexane). The reaction mixture was evaporated to dryness. The crude was purified by flash chromatography on silica gel (60–120 mesh) using 30% EtOAc-hexane as eluent to afford **34** as a white solid (170 mg, 75%). M.P. 158–160°C. ^1^H NMR (400 MHz, DMSO-d_6_): δ 12.88 (bs, 1H), 7.61 (dd, *J* = 8.0 Hz, 1H), 7.55–7.57 (m, 1H), 7.19 (d, *J* = 8.0 Hz, 1H), 7.08 (t, *J* = 7.4 Hz, 1H), 3.85 (s, 3H), 2.13 (bs, 3H); ^13^C NMR (100 MHz, DMSO-d_6_): δ 162.4, 157.1, 154.5, 146.6 (q, *J* = 32 Hz), 132.9, 130.4, 122.8, 121.9 (q, *J* = 275 Hz), 121.1, 120.5, 111.9, 55.9, 10.1 (q, *J* = 2.5 Hz). LCMS (ESI) m/z calculated for [C_13_H_11_F_3_N_2_O_2_ + H]^+^, 285.09; found 285.30.

### Synthesis of 5-Methyl-2-Phenyl-6-(Trifluoromethyl)Pyrimidin-4(3H)-One (35, 0496408)

Et_3_N (0.7 mL, 5.0 mmol) was added to a solution of ethyl 4,4,4-trifluoro-2-methyl-3-oxobutanoate **10** (200 mg, 1.0 mmol) and benzimidamide hydrochloride (120 mg, 1.0 mmol) in EtOH (6 mL) at RT. The reaction mixture was then heated at 80°C and monitored by TLC analysis (40% EtOAc-hexane) until completion. The reaction mixture was evaporated to dryness and poured to ice cooled water (20 mL). It was extracted with DCM (3 × 20 mL). The combined organic layer was dried over anhydrous Na_2_SO_4_ and concentrated under reduced pressure. The crude was further purified by column chromatography. The crude was further purified by column chromatography on silica gel (60–120 mesh) using 15% EtOAc-hexane as eluent to afford **35** as a white solid (135 mg, 52%). M.P. 256–259°C. ^1^H NMR (400 MHz, DMSO-d_6_): δ 13.29 (s, 1H), 8.10 (d, *J* = 6.1 Hz, 2H), 7.53–7.61 (m, 3H), 2.15 (s, 3H); ^13^C NMR (100 MHz, DMSO-d_6_): δ 163.5, 154.8, 146.5, 131.9, 131.4, 128.7, 127.7, 122.6, 121.8 (d, *J* = 274.5 Hz), 10.1. LCMS (ESI) m/z calculated for [C12H9F3N2O + H]+, 255.07; found 255.35.

### Synthesis of 2-(2-Fluorophenyl)-5-Methyl-6-(Trifluoromethyl)pyrimidin-4(3H)-One (36, 0496405)

Et_3_N (0.1 mL, 1.0 mmol) was added to a solution of 4,4,4-trifluoro-2-methyl-3-oxobutanoate (200 mg, 1.0 mmol) and 2-fluorobenzimidamide hydrochloride (170 mg, 1.0 mmol) in EtOH (3 mL) at RT. The reaction mixture was then heated at 80°C and monitored by TLC analysis (50% EtOAc-hexane) until completion. The reaction mixture was evaporated to dryness. The crude was purified by column chromatography on silica gel (60–120 mesh) using 25% EtOAc-hexane as eluent to afford **36** as a white solid (110 mg, 40%). M.P. 139–141°C. ^1^H NMR (400 MHz, DMSO-d_6_): δ 13.35 (s, 1H), 7.63–7.71 (m, 2H), 7.35–7.42 (m, 2H), 2.15 (s, 3H); ^13^C NMR (100 MHz, DMSO-d_6_): δ 162.8, 160.6, 158.2, 152.4, 146.4 (q, *J* = 37 Hz), 133.5 (d, *J* = 8.5 Hz), 124.7 (d, *J* = 3.5 Hz), 123.6, 121.7 (q, *J* = 274 Hz), 120.1 (d, *J* = 11.8 Hz), 116.3 (d, *J* = 21 Hz), 10.1 (q, J = 2.5 Hz). LCMS (ESI) m/z calculated for [C_12_H_8_F_4_N_2_O + H]^+^, 273.07; found 273.41.

### Synthesis of 2-(2-Chlorophenyl)-5-Methyl-6-(Trifluoromethyl)Pyrimidin-4(3H)-One (37, 0497560)

(i) Synthesis of 2-chlorobenzimidamide: 2-Chlorobenzonitrile (1.0 g, 7.3 mmol) was taken in anhydrous THF (20 mL) in a 50-mL round-bottom flask under N_2_ and cooled it down to −20°C. Dropwise lithium bis(trimethylsilyl)amide (1.06 M in THF) (8.2 mL, 8.8 mmol) was added to it. The reaction mixture was stirred at RT for 18 h and monitored by TLC analysis (50% EtOAc-hexane) until completion. 6N HCl (10 mL) was added to the reaction mixture and kept it stirring at for 30 min. The reaction mixture was then extracted with EtOAc (2 × 20 mL). The combined organic layer was rejected and the aqueous layer was basified by NaOH solution to pH 13–14. The aqueous layer was extracted with EtOAc (2 × 30 mL). The combined organic layer dried over anhydrous Na_2_SO_4_ and concentrated under reduced pressure to afford 2-chlorobenzimidamide as a pale yellow-colored solid (550 mg, 49%). ^1^H NMR (400 MHz, DMSO-d_6_): δ 7.44–7.47 (m, 1H), 7.32–7.39 (m, 3H), 6.33 (bs, 3H). LCMS (ESI) m/z calculated for [C_7_H_7_ClN_2_ + H]^+^, 155.04; found 155.01. (ii) Et_3_N (0.1 mL, 0.7 mmol) was added to a solution of ethyl 4,4,4-trifluoro-2-methyl-3-oxobutanoate (150 mg, 0.7 mmol) and 2-chlorobenzimidamide (120 mg, 0.7 mmol) in EtOH (5 mL) at RT. The reaction mixture was then heated at 80°C and monitored by TLC analysis (80% EtOAc-hexane) until completion. The reaction mixture was evaporated to dryness. The crude was purified by flash chromatography on silica gel (60–120 mesh) using 30% EtOAc-hexane as eluent to afford **37** as a white solid (170 mg, 77%). M.P. 165–167°C. ^1^H NMR (400 MHz, DMSO-d_6_): δ 13.44 (s, 1H), 7.56–7.64 (m, 3H), 7.50 (t, *J* = 7.3 Hz, 1H), 2.16 (bs, 3H); ^13^C NMR (100 MHz, DMSO-d_6_): δ 162.7, 154.6, 146.2 (q, *J* = 38 Hz), 132.3, 132.2, 131.4, 130.9, 129.7, 127.3, 124.0, 121.8 (q, *J* = 274 Hz), 10.1 (d, *J* = 2.1 Hz). LCMS (ESI) m/z calculated for [C_12_H_8_ClF_3_N_2_O + H]^+^, 289.04; found 289.26.

### Synthesis of 2-(3-Chloropyridin-2-yl)-5-Methyl-6-(Trifluoromethyl)Pyrimidin-4(3H)-One (38)

(i) Synthesis of 3-chloropicolinimidamide: Chloropicolinonitrile (500 mg, 3.6 mmol) was taken in anhydrous THF (20 mL) in a 50-mL round-bottom flask under N_2_ and cooled it down to −20°C. Dropwise lithium bis(trimethylsilyl)amide (1.06 M in THF) (10.2 mL, 10.8 mmol) was added to it. The reaction mixture was stirred at RT and monitored by TLC analysis (50% EtOAc-hexane) until completion. 6N HCl (10 mL) was added to the reaction mixture and kept it stirring at for 30 min. The reaction mixture was then extracted with EtOAc (2 × 20 mL). The combined organic layer was rejected and the aqueous layer was basified by NaOH solution to pH 13–14. The aqueous layer was extracted with EtOAc (2 × 30 mL). The combined organic layer dried over anhydrous Na_2_SO_4_ and concentrated under reduced pressure to afford 3-chloropicolinimidamide as a brown-colored solid (350 mg, 62%). ^1^H NMR (400 MHz, DMSO-d_6_): δ 8.50 (d, *J* = 4.6 Hz, 1H), 7.97 (d, *J* = 8.1 Hz, 1H), 7.45 (q, *J* = 4.6 Hz, 1H), 6.50 (bs, 3H). (ii) Et_3_N (0.3 mL, 1.9 mmol) was added to a solution of ethyl 4,4,4-trifluoro-2-methyl-3-oxobutanoate (220 mg, 1.1 mmol) and 3-chloropicolinimidamide (150 mg, 0.9 mmol) in EtOH (5 mL) at RT. The reaction mixture was then heated at 80°C and monitored by TLC analysis (70% EtOAc-hexane) until completion. The reaction mixture was evaporated to dryness. The crude was purified by flash chromatography on silica gel (60–120 mesh) using 25% EtOAc-hexane as eluent to afford **38** as a white solid (100 mg, 35%). M.P. 152–153°C. ^1^H NMR (400 MHz, DMSO-d_6_): δ 13.49 (s, 1H), 8.67 (d, *J* = 4.5 Hz, 1H), 8.17 (d, *J* = 8.2 Hz, 1H), 7.66 (t, *J* = 4.6 Hz, 1H), 2.18 (bs, 3H); ^13^C NMR (100 MHz, DMSO-d_6_): δ 162.5, 153.1, 147.7, 147.6, 145.8 (q, *J* = 33 Hz), 138.8, 129.9, 127.0, 125.2, 121.7 (q, *J* = 274 Hz), 10.2 (q, *J* = 2.2 Hz). LCMS (ESI) m/z calculated for [C_11_H_7_ClF_3_N_3_O + H]^+^, 290.03; found 290.25.

### Synthesis of 2-(3-Methoxypyridin-2-yl)-5-Methyl-6-(Trifluoromethyl)Pyrimidin-4(3H)-One (39, 0499223)

(i) Synthesis of 3-methoxypicolinimidamide: 3-Methoxypicolinonitrile (1.0 g, 7.4 mmol) was taken in anhydrous THF (10 mL) in a 50-mL round-bottom flask under N_2_ and cooled it down to −20°C. Dropwise lithium bis(trimethylsilyl)amide (1.06 M in THF) (21 mL, 22.4 mmol) was added to it. The reaction mixture was stirred at RT and monitored by TLC analysis (50% EtOAc-hexane) until completion. 6N HCl (20 mL) was added to the reaction mixture and kept it stirring at for 30 min. The reaction mixture was then extracted with EtOAc (2 × 20 mL). The combined organic layer was rejected and the aqueous layer was basified by NaOH solution to pH 13–14. The aqueous layer was extracted with EtOAc (2 × 30 mL). The combined organic layer dried over anhydrous Na_2_SO_4_ and concentrated under reduced pressure. It was further recrystallized from ether-pentane to afford 3-methoxypicolinimidamide as a pale brown solid (700 mg, 62%). ^1^H NMR (400 MHz, DMSO-d_6_): δ 8.21 (bs, 1H), 7.63–7.65 (m, 1H), 7.42–7.53 (m, 2H), 7.03 (bs, 2H), 3.91 (s, 3H). (ii) Et_3_N (0.4 mL, 2.6 mmol) was added to a solution of ethyl 4,4,4-trifluoro-2-methyl-3-oxobutanoate (310 mg, 1.5 mmol) and 3-methoxypicolinimidamide (200 mg, 1.3 mmol) in EtOH (5 mL) at RT. The reaction mixture was then heated at 80°C and monitored by TLC analysis (5% MeOH–DCM) until completion. The reaction mixture was evaporated to dryness. The crude was purified by flash chromatography on silica gel (60–120 mesh) using 2% MeOH–DCM as eluent to afford **39** as an off-white solid (80 mg, 21%). M.P. 164–165°C. ^1^H NMR (400 MHz, DMSO-d_6_): δ 13.22 (s, 1H), 8.26 (d, *J* = 3.7 Hz, 1H), 7.69 (d, *J* = 8.1 Hz, 1H), 7.58–7.61 (m, 1H), 3.85 (s, 3H), 2.16 (bs, 3H); ^13^C NMR (100 MHz, DMSO-d_6_): δ 162.4, 154.2, 153.5, 146.4 (q, *J* = 34 Hz), 140.8, 139.5, 126.9, 124.3, 121.8 (q, *J* = 275 Hz), 120.5, 56.1, 10.2 (q, *J* = 2.4 Hz). LCMS (ESI) m/z calculated for [C_12_H_10_F_3_N_3_O_2_ + H]^+^, 286.08; found 286.29.

### Synthesis of 2-Cyclohexyl-5-Methyl-6-(Trifluoromethyl)Pyrimidin-4(3H)-One (40, 0504095)

(i) Synthesis of N′-hydroxycyclohexanecarboximidamide: Hydroxylamine (1.9 g, 27.5 mmol) was taken in EtOH (20 mL) in a 100-mL round-bottom flask under N_2_. Cyclohexanecarbonitrile (2.0 g, 18.3 mmol) and Et_3_N (3.3 mL, 22.9 mmol) were sequentially added to it. The reaction mixture was refluxed and monitored by TLC analysis (50% EtOAc-hexane) until completion. The reaction mixture was concentrated under reduced pressure and the crude was purified by flash chromatography on silica gel (60–120 mesh) using 20% EtOAc-hexane as eluent to afford N′-hydroxycyclohexanecarboximidamide as a gray solid (820 mg, 31%). ^1^H NMR (400 MHz, DMSO-d_6_): δ 8.67 (s, 1H), 5.21 (bs, 2H), 1.92–1.98 (m, 1H), 1.62–1.80 (m, 4H), 1.50–1.59 (m, 1H), 1.35–1.38 (m, 2H), 1.13–1.24 (m, 3H). (ii) Synthesis of cyclohexanecarboximidamide: A par flask was charged with N′-hydroxycyclohexanecarboximidamide (1.5 g, 11.1 mmol) and MeOH (20 mL) followed by addition of Pd-C (20% wt/wt, 0.6 g). The flask was evacuated under vacuum and then purged with hydrogen. The reaction was stirred under hydrogen atmosphere (60 psi) for 24 h. The reaction was monitored by TLC. It was then filtered through sintered funnel with a pad of celite, washed with MeOH (30 mL) and concentrated under reduced pressure. It was recrystallized from diethyl ether/pentane to afford cyclohexanecarboximidamide as a pale brown solid (0.56 g, 77%). MS m/z 127.2 [M^−^+ H]. (iii) Et_3_N (0.6 mL, 4.4 mmol) was added to a solution of ethyl 4,4,4-trifluoro-2-methyl-3-oxobutanoate (520 mg, 2.6 mmol) and cyclohexanecarboximidamide (280 mg, 2.2 mmol) in EtOH (5 mL) at RT. The reaction mixture was then heated at 80°C and monitored by TLC analysis (50% EtOAc-hexane) until completion. The reaction mixture was evaporated to dryness. The crude was purified by flash chromatography on silica gel (60–120 mesh) using 25% EtOAc-hexane as eluent to afford **40** as a white solid (120 mg, 21%). M.P. 169–170°C. ^1^H NMR (400 MHz, DMSO-d_6_): δ 12.84 (s, 1H), 3.22 (s, 3H), 2.54–2.57 (m, 1H), 1.74–1.83 (m, 4H), 1.63–1.66 (m, 1H), 1.43–1.52 (m, 2H), 1.17–1.31 (m, 3H); ^13^C NMR (100 MHz, DMSO-d_6_): δ 163.6, 163.0, 146.3 (d, *J* = 30 Hz), 123.2 (q, *J* = 274 Hz), 121.9, 42.3, 29.9, 25.2, 25.1, 9.8. LCMS (ESI) m/z calculated for [C_12_H_15_F_3_N_2_O + H]^+^, 261.12; found 261.07.

### Synthesis of 5-Methyl-6-(Trifluoromethyl)-[2,2′-Bipyrimidin]-4(3H)-One (41, 0504071)

(i) Synthesis of pyrimidine-2-carboximidamide: Pyrimidine-2-carbonitrile (500 mg, 4.7 mmol) was taken in anhydrous THF (10 mL) in a 50-mL round-bottom flask under N_2_ and cooled it down to −20°C. Dropwise lithium bis(trimethylsilyl)amide (1.06 M in THF) (5.3 mL, 5.7 mmol) was added to it. The reaction mixture was stirred at RT for 18 h and monitored by TLC analysis (50% EtOAc-hexane). 6N HCl (10 mL) was added to the reaction mixture and kept it stirring at for 30 min. The reaction mixture was then extracted with EtOAc (2 × 20 mL). The combined organic layer was rejected and the aqueous layer was basified by NaOH solution to pH 13–14. The aqueous layer was extracted with EtOAc (2 × 30 mL). The combined organic layer dried over anhydrous Na_2_SO_4_ and concentrated under reduced pressure. It was further recrystallized from diethyl ether-pentane to afford pyrimidine-2-carboximidamide as a pale yellow solid (420 mg, 72%). ^1^H NMR (400 MHz, DMSO-d_6_): δ 8.95 (d, *J* = 4.8 Hz, 2H), 7.62–7.67 (m, 1H), 7.14 (bs, 3H). (ii) Et_3_N (0.5 mL, 3.2 mmol) was added to a solution of ethyl 4,4,4-trifluoro-2-methyl-3-oxobutanoate **10** (380 mg, 1.9 mmol) and pyrimidine-2-carboximidamide (200 mg, 1.6 mmol) in EtOH (5 mL) at RT. The reaction mixture was then heated at 80°C and monitored by TLC analysis (80% EtOAc-hexane) until completion. The reaction mixture was evaporated to dryness. The crude was purified by flash chromatography on silica gel (60–120 mesh) using 50% EtOAc-hexane as eluent to afford **41** as a white solid (120 mg, 28%). M.P. 229–230°C. ^1^H NMR (400 MHz, DMSO-d_6_): δ 13.16 (s, 1H), 9.06 (d, *J* = 4.9 Hz, 2H), 7.75 (t, *J* = 4.9 Hz, 1H), 2.19 (bs, 3H); ^13^C NMR (100 MHz, DMSO-d_6_): δ 162.5, 158.1, 156.8, 151.9, 146.2 (q, *J* = 36 Hz), 126.4, 123.1, 121.8 (q, *J* = 275 Hz), 10.5 (q, *J* = 2.0 Hz). LCMS (ESI) m/z calculated for [C_10_H_7_F_3_N_4_O + H]^+^, 257.07; found 257.2.

### Synthesis of 5-Methyl-6-(Trifluoromethyl)-2-(2-(Trifluoromethyl)Phenyl)pyrimidin-4(3H)-One (42, 0498344)

(i) Synthesis of 2-(trifluoromethyl)benzimidamide: 2-(trifluoromethyl)benzonitrile 61 (500 mg, 2.9 mmol) was taken in anhydrous THF (10 mL) in a 50-mL round-bottom flask under N_2_ and cooled it down to −20°C. Dropwise lithium bis(trimethylsilyl)amide (1.06 M in THF) (2.7 mL, 2.9 mmol) was added to it. The reaction mixture was stirred at RT and monitored by TLC analysis (50% EtOAc-hexane) until completion. 6N HCl (10 mL) was added to the reaction mixture and kept it stirring at for 30 min. The reaction mixture was then extracted with EtOAc (2 × 20 mL). The combined organic layer was rejected and the aqueous layer was basified by NaOH solution to pH 13–14. The aqueous layer was extracted with EtOAc (2 × 30 mL). The combined organic layer dried over anhydrous Na_2_SO_4_ and concentrated under reduced pressure to afford 2-(trifluoromethyl)benzimidamide as a pale brown solid (300 mg, 54%). ^1^H NMR (400 MHz, DMSO-d_6_): δ 7.73 (d, *J* = 7.8 Hz, 1H), 7.67 (t, *J* = 7.5 Hz, 1H), 7.57 (t, *J* = 7.6 Hz, 1H), 7.46 (d, *J* = 7.2 Hz, 1H), 6.38 (bs, 3H). (ii) Et_3_N (0.2 mL, 1.6 mmol) was added to a solution of ethyl 4,4,4-trifluoro-2-methyl-3-oxobutanoate (189 mg, 0.9 mmol) and 2-(trifluoromethyl)benzimidamide (150 mg, 0.8 mmol) in EtOH (5 mL) at RT. The reaction mixture was then heated at 80°C and monitored by TLC analysis (50% EtOAc-hexane) until completion. The reaction mixture was evaporated to dryness. The crude was purified by flash chromatography on silica gel (60–120 mesh) using 30% EtOAc-hexane as eluent to afford **42** as an off-white solid (130 mg, 50%). M.P. 168–170°C. ^1^H NMR (400 MHz, DMSO-d_6_): δ 13.52 (s, 1H), 7.91 (d, *J* = 7.5 Hz, 1H), 7.75–7.85 (m, 3H), 2.16 (bs, 3H); ^13^C NMR (100 MHz, DMSO-d_6_): δ 162.8, 154.9, 145.9 (q, *J* = 27 Hz), 132.5, 131.6, 131.1, 130.8, 126.6 (q, *J* = 4.6 Hz), 126.8 (q, *J* = 31 Hz), 119.9 (q, *J* = 274 Hz), 125.4 (q, *J* = 274 Hz), 123.9, 10.1 (q, *J* = 2.1 Hz). LCMS (ESI) m/z calculated for [C_13_H_8_F_6_N_2_O + H]^+^, 323.06; found 323.28.

### Synthesis of 2-(2,6-Dimethylphenyl)-5-Methyl-6-(Trifluoromethyl)Pyrimidin-4(3H)-One (43, 0504067)

(i) Synthesis of N-hydroxy-2,6-dimethylbenzimidamide: Hydroxylamine (1.59 g, 22.9 mmol) was taken in EtOH (20 mL) in a 100-mL round-bottom flask under N_2_. 2,6-Dimethylbenzonitrile 105 (2.0 g, 15.2 mmol) and Et_3_N (3.3 mL, 22.9 mmol) were sequentially added to it. The reaction mixture was refluxed and monitored by TLC analysis (50% EtOAc-hexane) until completion. The reaction mixture was concentrated under reduced pressure and the crude was purified by flash chromatography on silica gel (60–120 mesh) using 20% EtOAc-hexane as eluent to afford N-hydroxy-2,6-dimethylbenzimidamide as a gray solid (600 mg, 24%). LC-MS: 165.07 (M+H^+^); 98.79% (purity). (ii) Synthesis of 2,6-dimethylbenzimidamide: A par flask was charged with N-hydroxy-2,6-dimethylbenzimidamide (600 mg, 3.6 mmol) and MeOH (20 mL) followed by addition of Pd-C (20% wt/wt, 300 mg). The flask was evacuated under vacuum and then purged with hydrogen. The reaction was stirred under hydrogen atmosphere (60 psi) at RT for 48 h. The reaction was monitored by TLC. It was then filtered through sintered funnel with a pad of celite, washed with MeOH (30 mL) and concentrated under reduced pressure to afford 2,6-dimethylbenzimidamide as an off-white solid (550 mg, crude). This was then used in the next step without further purification. ^1^H NMR (400 MHz, DMSO-d_6_): δ 7.83 (s, 1H), 7.46 (s, 1H), 7.11–7.15 (m, 2H), 7.01–7.03 (m, 2H), 2.24 (s, 6H). (iii) Et_3_N (0.6 mL, 4.0 mmol) was added to a solution of ethyl 4,4,4-trifluoro-2-methyl-3-oxobutanoate (480 mg, 2.4 mmol) and 2,6-dimethylbenzimidamide (300 mg, 2.0 mmol) in EtOH (10 mL) at RT. The reaction mixture was then heated at 80°C for 18 h and monitored by TLC analysis (50% EtOAc-hexane). The reaction mixture was evaporated to dryness. The crude was purified by flash chromatography on silica gel (60–120 mesh) using 20% EtOAc-hexane as eluent to afford **43** as a white solid (50 mg, 9%). M.P. 174–175°C. ^1^H NMR (400 MHz, DMSO-d_6_): δ ^1^H NMR (400 MHz, DMSO-d_6_): δ 13.24 (s, 1H), 7.31 (t. *J* = 7.6 Hz, 1H), 7.16 (d. *J* = 7.6 Hz, 2H), 2.15 (s, 3H), 2.13 (s, 6H); ^13^C NMR (100 MHz, DMSO-d_6_): δ 162.8, 156.5, 146.1 (q, *J* = 32 Hz), 135.6, 133.1, 129.6, 127.4, 121.8 (q, *J* = 274 Hz), 18.9, 10.0 (q, *J* = 3.0 Hz). LCMS (ESI) m/z calculated for [C_14_H_13_F_3_N_2_O + H]^+^, 283.11; found 283.26.

### Synthesis of 5-Methyl-2-(2-(Trifluoromethoxy)Phenyl)-6-(Trifluoromethyl)Pyrimidin-4(3H)-One (44, 0504066)

(i) Synthesis of 2-(trifluoromethoxy)benzimidamide: 2-(Trifluoromethoxy)benzonitrile (500 mg, 2.7 mmol) was taken in anhydrous THF (10 mL) in a 50-mL round-bottom flask under N_2_ and cooled it down to −20°C. Dropwise lithium bis(trimethylsilyl)amide (1.06 M in THF) (5.1 mL, 5.4 mmol) was added to it. The reaction mixture was stirred at RT for 18 h and monitored by TLC analysis (50% EtOAc-hexane). 6N HCl (20 mL) was added to the reaction mixture and kept it stirring at for 30 min. The reaction mixture was then extracted with EtOAc (2 × 20 mL). The combined organic layer was rejected and the aqueous layer was basified by NaOH solution to pH 13–14. The aqueous layer was extracted with EtOAc (2 × 30 mL). The combined organic layer dried over anhydrous Na_2_SO_4_ and concentrated under reduced pressure. It was further recrystallized from ether-pentane to afford 2-(trifluoromethoxy)benzimidamide as a pale brown solid (470 mg, 87%). ^1^H NMR (400 MHz, DMSO-d_6_): δ 7.49–7.53 (m, 2H), 7.38–7.43 (m, 2H), 6.45 (bs, 3H). (ii) Et_3_N (0.3 mL, 1.9 mmol) was added to a solution of ethyl 4,4,4-trifluoro-2-methyl-3-oxobutanoate (230 mg, 1.2 mmol) and 2-(trifluoromethoxy)benzimidamide (200 mg, 0.9 mmol) in EtOH (5 mL) at RT. The reaction mixture was then heated at 80°C for 18 h and monitored by TLC analysis (50% EtOAc-hexane). The reaction mixture was evaporated to dryness. The crude was purified by flash chromatography on silica gel (60–120 mesh) using 20% EtOAc-hexane as eluent to afford **44** as a white solid (205 mg, 62%). M.P. 167–168°C. ^1^H NMR (400 MHz, DMSO-d_6_): δ ^1^H NMR (400 MHz, DMSO-d_6_): δ 13.46 (s, 1H), 7.70–7.78 (m, 2H), 7.55–7.58 (m, 2H), 2.16 (d. *J* = 2.1 Hz); ^13^C NMR (100 MHz, DMSO-d_6_): δ 162.7, 152.7, 145.6, 132.9, 131.5, 127.7, 126.6, 123.7, 121.5 (q, *J* = 274 Hz), 121.2, 118.6, 116.1, 10.1 (d, *J* = 2.5 Hz). LCMS (ESI) m/z calculated for [C_13_H_8_F_6_N_2_O_2_ + H]^+^, 339.22; found 339.20.

### Synthesis of 2-(3-Fluoropyridin-2-yl)-5-Methyl-6-(Trifluoromethyl)Pyrimidin-4(3H)-One (45, 0504056)

(i) Synthesis of 3-fluoropicolinimidamide: 3-Fluoropicolinonitrile (2.0 g, 16.4 mmol) was taken in anhydrous THF (20 mL) in a 100-mL round-bottom flask under N_2_ and cooled it down to −20°C. Dropwise lithium bis(trimethylsilyl)amide (1.06 M in THF) (30.9 mL, 32.8 mmol) was added to it. The reaction mixture was stirred at RT and monitored by TLC analysis (50% EtOAc-hexane) until completion. 6N HCl (30 mL) was added to the reaction mixture and kept it stirring at for 30 min. The reaction mixture was then extracted with EtOAc (2 × 20 mL). The combined organic layer was rejected and the aqueous layer was basified by NaOH solution to pH 13–14. The aqueous layer was extracted with EtOAc (2 × 30 mL). The combined organic layer dried over anhydrous Na_2_SO_4_ and concentrated under reduced pressure. It was further recrystallized from ether-pentane to afford 3-fluoropicolinimidamide as a brown solid (900 mg, 40%). MS: 139.91 (M+H)^+^. (ii) Et_3_N (4.7 mL, 32.3 mmol) was added to a solution of ethyl 4,4,4-trifluoro-2-methyl-3-oxobutanoate (1.28 g, 6.5 mmol) and 3-fluoropicolinimidamide (900 mg, 6.5 mmol) in EtOH (20 mL) at RT. The reaction mixture was then heated at 80°C and monitored by TLC analysis (50% EtOAc-hexane) until completion. The reaction mixture was evaporated to dryness. The crude was purified by flash chromatography on silica gel (60–120 mesh) using 24% EtOAc-hexane as eluent to afford **45** as a white solid (200 mg, 11%). M.P. 173–175°C. ^1^H NMR (400 MHz, DMSO-d_6_): δ 13.19 (s, 1H), 8.60 (d, *J* = 4.5 Hz, 1H), 7.98 (t, *J* = 8.9 Hz, 1H), 7.73–7.77 (m, 1H), 2.17 (bs, 3H); ^13^C NMR (100 MHz, DMSO-d_6_): δ 162.4, 157.7 (d, *J* = 265 Hz), 151.2, 145.9 (q, *J* = 33 Hz), 145.4, 137.2 (d, *J* = 8.7 Hz), 128.5 (d, *J* = 4.9 Hz), 126.0 (d, *J* = 19 Hz), 125.3, 121.7 (q, *J* = 275 Hz), 10.3 (q, *J* = 2.5 Hz). LCMS (ESI) m/z calculated for [C_11_H_7_F_4_N_3_O + H]^+^, 274.06; found 274.14.

### Synthesis of 2-(2-Hydroxyphenyl)-5-Methyl-6-(Trifluoromethyl)Pyrimidin-4(3H)-One (46, 0499007)

Et_3_N (0.4 mL, 3.0 mmol) was added to a solution of ethyl 4,4,4-trifluoro-2-methyl-3-oxobutanoate **8** (349 mg, 1.8 mmol) and 2-hydroxybenzimidamide (200 mg, 1.5 mmol) in EtOH (5 mL) at RT. The reaction mixture was then heated at 80°C and monitored by TLC analysis (50% EtOAc-hexane) until completion. The reaction mixture was evaporated to dryness. The crude was purified by flash chromatography on silica gel (60–120 mesh) using 30% EtOAc-hexane as eluent to afford **46** as a pale yellow solid (120 mg, 30%). M.P. 270–271°C. ^1^H NMR (400 MHz, DMSO-d_6_): δ 12.91–12.27 (bs, 2H), 8.08 (d, *J* = 7.8 Hz, 1H), 7.45 (t, *J* = 8.4 Hz, 1H), 7.01–6.96 (m, 2H), 2.15 (bs, 3H); ^13^C NMR (100 MHz, DMSO-d_6_): δ 163.2, 158.6, 155.7, 145.2, 133.9, 128.5, 121.6 (q, *J* = 276 Hz), 119.3, 117.6, 114.8, 10.1 (q, *J* = 1.7 Hz). LCMS (ESI) m/z calculated for [C_12_H_9_F_3_N_2_O_2_ + H]^+^, 271.07; found 271.03.

### Synthesis of 6-Benzyl-5-Methyl-2-(Pyridin-2-yl)Pyrimidin-4(3H)-One (47, 0497408)

**(**i) Synthesis of ethyl 2-methyl-3-oxo-4-phenylbutanoate: Ethyl 3-oxo-4-phenylbutanoate (450 mg, 2.2 mmol) was taken in DMF (10 mL) in a 50-mL round-bottom flask under N_2_. Iodomethane (0.1 mL, 1.5 mmol) and K_2_CO_3_ (300 mg, 2.2 mmol) were sequentially added to it. The reaction mixture was stirred at RT for 2 h. The reaction mixture was then poured into ice water (20 g) and extracted with EtOAc (2 × 20 mL). The combined organic layer was dried over anhydrous Na_2_SO_4_ and concentrated under reduced pressure to generate a colorless liquid (0.1 g, crude) which was then used as such for the next step without further purification. LC-MS: 219.17 [M-H^+^]; 50.74% (purity). (ii) Na_2_CO_3_ (40 mg, 0.4 mmol) was added to a solution of ethyl 2-methyl-3-oxo-4-phenylbutanoate (100 mg, 0.4 mmol) and picolinimidamide hydrochloride (70 mg, 0.4 mmol) in EtOH (3 mL) at RT. The reaction mixture was then heated at 80°C for 4 h and monitored by TLC analysis (50% EtOAc-hexane) until completion. The reaction mixture was evaporated to dryness. The crude was purified by flash chromatography on silica gel (60–120 mesh) using 30% EtOAc-hexane as eluent to afford **47** as a white solid (25 mg, 20%). M.P. 120–121°C. ^1^H NMR (400 MHz, DMSO-d_6_): δ 11.95 (s, 1H), 8.71 (d, *J* = 4.4 Hz, 1H), 8.26 (d, *J* = 7.9 Hz, 1H), 8.00–8.04 (m, 1H), 7.61 (q, *J* = 5.1 Hz, 1H), 7.28–7.34 (m, 4H), 7.20 (t, *J* = 6.8 Hz, 1H), 3.99 (s, 2H), 2.06 (s, 3H); ^13^C NMR (100 MHz, DMSO-d_6_): δ 162.1, 160.4, 150.9, 149.1, 148.5, 138.2, 137.9, 128.7, 128.4, 126.4, 126.3, 121.9, 120.8, 40.3, 11.0. LCMS (ESI) m/z calculated for [C_17_H_15_N_3_O + H]^+^, 278.13; found 278.32.

### Synthesis of 5-Methyl-6-Phenyl-2-(Pyridin-2-yl)Pyrimidin-4(3H)-One (48, 0496303)

Synthesis of ethyl 2-methyl-3-oxo-3-phenylpropanoate: Ethyl 3-oxo-3-phenylpropanoate (1.0 g, 5.2 mmol) was taken in DMF (5 mL) in a 50-mL round-bottom flask under N_2_. Iodomethane (0.4 mL, 6.2 mmol) and K_2_CO_3_ (1.4 g, 10.4 mmol) were sequentially added to it. The reaction mixture was heated at 60°C for 5 h. The reaction mixture was then poured into ice water (20 g) and extracted with EtOAc (2 × 20 mL). The combined organic layer was dried over anhydrous Na_2_SO_4_ and concentrated under reduced pressure. The crude was purified by column chromatography on silica gel (100–200 mesh) using 5% EtOAc-hexane as eluent to afford ethyl 2-methyl-3-oxo-3-phenylpropanoate as a brown liquid (0.61 g, 57%). ^1^H NMR (400 MHz, CDCl_3_): δ 7.98 (d, *J* = 7.32 Hz, 2H), 7.57 (d, *J* = 7.52 Hz, 1H), 7.51 (t, *J* = 7.60 Hz, 2H), 4.37 (q, *J* = 7.10 Hz, 1H), 4.19–4.21 (m, 2H), 1.49 (d, *J* = 7.10 Hz, 3H) and 1.16 (t, *J* = 7.1 Hz, 3H). (ii) Na_2_CO_3_ (436 mg, 4.4 mmol) was added to a solution of ethyl 2-methyl-3-oxo-3-phenylpropanoate (600 mg, 2.9 mmol) and picolinimidamide hydrochloride (352 mg, 2.9 mmol) in EtOH (3 mL) at RT. The reaction mixture was then heated at 90°C and monitored by TLC analysis (Hexane/EtOAc = 1:1) until completion. The reaction mixture was evaporated to dryness and poured to ice cooled water (20 mL). It was extracted with EtOAc (2 × 20 mL). The combined organic layer was dried over anhydrous Na_2_SO_4_ and concentrated under reduced pressure. The crude was further purified by column chromatography on silica gel (60–120 mesh) using 20% hexane-EtOAc as eluent to afford **48** as a white solid (230 mg, 30%). M.P. 140–141°C. ^1^H NMR (400 MHz, DMSO-d_6_): δ 12.15 (s, 1H), 8.74 (d, *J* = 4.5 Hz, 1H), 8.32 (d, *J* = 7.9 Hz, 1H), 8.02 (t, *J* = 7.6 & 6.7 Hz, 1H), 7.62–7.67 (m, 3H), 7.46–7.53 (m, 3H), 2.08 (s, 3H); ^13^C NMR (100 MHz, DMSO-d_6_): δ 162.5, 158.4, 151.0, 149.0, 148.6, 138.3, 137.9, 128.9, 128.8, 128.1, 126.4, 122.1, 120.7, 12.9. LCMS (ESI) m/z calculated for [C_16_H_13_N_3_O + H]^+^, 264.11; found 264.33.

### Synthesis of 5,6-Dimethyl-2-(Pyridin-2-yl)Pyrimidin-4(3H)-One (49, 0496407)

Et_3_N (2.4 mL, 17.5 mmol) was added to a solution of ethyl 2-methyl-3-oxobutanoate (500 mg, 3.5 mmol) and picolinimidamide hydrochloride (462 mg, 3.8 mmol) in EtOH (10 mL) at RT. The reaction mixture was then heated at 120°C and monitored by TLC analysis (50% EtOAc-hexane) until completion. The reaction mixture was evaporated to dryness. The crude was further purified by column chromatography on silica gel (60–120 mesh) using 15% EtOAc-hexane as eluent to afford **49** as an off-white solid (80 mg, 11%). M.P. 146°C. ^1^H NMR (400 MHz, DMSO-d_6_): δ 11.88 (s, 1H), 8.71 (d, *J* = 4.3 Hz, 1H), 8.28 (d, *J* = 7.9 Hz, 1H), 8.02 (t, *J* = 7.7 Hz, 1H), 7.60–7.63 (m, 1H), 2.32 (s, 3H), 1.99 (s, 3H); ^13^C NMR (100 MHz, DMSO-d_6_): δ 161.6, 158.8, 150.5, 149.0, 148.5, 137.9, 126.3, 121.8, 120.3, 21.6, 11.0. LCMS (ESI) m/z calculated for [C_11_H_11_N_3_O + H]^+^, 202.10; found 202.42

### Synthesis of 5-Methyl-2-(Pyridin-2-yl)Pyrimidin-4(3H)-One (50, 0496336)

(i) Synthesis of ethyl 2-methyl-3-oxopropanoate: Ethyl 3-oxopropanoate (1.0 g, 8.6 mmol) was taken in DMF (15 mL) in a 50-mL round-bottom flask under N_2_. Iodomethane (0.8 mL, 12.9 mmol) and K_2_CO_3_ (2.4 g, 17.2 mmol) were sequentially added to it. The reaction mixture was heated at 60°C for 5 h. The reaction mixture was then poured into ice water (20 g) and extracted with EtOAc (2 × 20 mL). The combined organic layer was dried over anhydrous Na_2_SO_4_ and concentrated under reduced pressure. The crude was purified by column chromatography on silica gel (100–200 mesh) using 5% EtOAc-hexane as eluent to afford ethyl 2-methyl-3-oxopropanoate as a brown liquid (0.61 g, 54%). MS m/z 131.1 [M^−^+ H]. (ii) Na_2_CO_3_ (330 mg, 3.2 mmol) was added to a solution of ethyl 2-methyl-3-oxo-propanoate (220 mg, 1.7 mmol) and picolinimidamide hydrochloride (250 mg, 1.6 mmol) in EtOH (5 mL) at RT. The reaction mixture was then heated at 90°C and monitored by TLC analysis (Hexane/EtOAc = 1:1) until completion. The reaction mixture was evaporated to dryness and poured to ice cooled water (20 mL). It was extracted with EtOAc (2 × 20 mL). The combined organic layer was dried over anhydrous Na_2_SO_4_ and concentrated under reduced pressure. The crude was further purified by column chromatography on silica gel (60–120 mesh) using 2% MeOH–DCM as eluent to afford **50** as an off-white solid (110 mg, 38%). M.P. 148–149°C. ^1^H NMR (400 MHz, DMSO-d_6_): δ 11.04 (s, 1H), 8.64 (d, *J* = 4.7 Hz, 1H), 8.37 (d, *J* = 7.9 Hz, 1H), 7.86–7.92 (m, 2H), 7.44–7.47 (m, 1H), 2.15 (s, 3H); ^13^C NMR (100 MHz, DMSO-d_6_): δ 162.2, 152.2, 151.3, 148.9, 147.9, 137.7, 126.3, 126.1, 121.7, 13.4. LCMS (ESI) m/z calculated for [C_10_H_9_N_3_O + H]^+^, 188.07; found 188.08.

### Synthesis of 5-Methyl-2-(Pyridin-2-yl)-6-(Trifluoromethyl)Pyrimidin-4(3H)-One (51, 0340734)

Et_3_N (0.7 mL, 5.0 mmol) was added to a solution of ethyl 4,4,4-trifluoro-2-methyl-3-oxobutanoate (200 mg, 1.0 mmol) and picolinimidamide hydrochloride (160 mg, 1.0 mmol) in EtOH (5 mL) at RT. The reaction mixture was then heated at 80°C for 12 h and monitored by TLC analysis (50% EtOAc-hexane) until completion. The reaction mixture was evaporated to dryness and poured to ice cooled water (20 mL). It was extracted with DCM (3 × 20 mL). The combined organic layer was dried over anhydrous Na_2_SO_4_ and concentrated under reduced pressure. The crude was further purified by column chromatography on silica gel (60–120 mesh) using 20% EtOAc-hexane as eluent to afford **51** as a white solid (150 mg, 58%). M.P. 153–155°C. ^1^H NMR (400 MHz, DMSO-d_6_): δ 11.82 (s, 1H), 8.76 (d, *J* = 4.3 Hz, 1H), 8.28 (d, *J* = 7.9 Hz, 1H), 8.07 (t, *J* = 7.7 Hz, 1H), 7.65–7.68 (m, 1H), 2.17 (d, *J* = 2.2 Hz, 3H); ^13^C NMR (100 MHz, DMSO-d_6_): δ 162.2, 152.7, 149.2, 147.8, 145.9 (q, *J* = 32 Hz), 138.0, 126.9, 125.0, 122.4, 121.8 (q, *J* = 274 Hz), 10.3 (q, *J* = 2.3 Hz). LCMS (ESI) m/z calculated for [C_11_H_8_F_3_N_3_O + H]^+^, 256.07; found 256.32.

### Synthesis of 5-Benzyl-2-(Pyridin-2-yl)-6-(Trifluoromethyl)Pyrimidin-4(3H)-One (52, 0504059)

(i) Synthesis of ethyl 2-benzyl-4,4,4-trifluoro-3-oxobutanoate: 3-Phenylpropanoyl chloride (1.0 g, 5.9 mmol) was taken in DCM (10 mL) in a 100-mL round-bottom flask under N_2_. TFAA (1.3 mL, 8.9 mmol) and pyridine (1.0 mL, 11.8 mmol) were sequentially added to it. The reaction mixture was stirred at RT for 3 h. EtOH (2.5 mL) was added to the reaction mixture and kept it stirring for another 12 h. The reaction mixture was concentrated under reduced pressure and triturated with hexane. This afforded ethyl 2-benzyl-4,4,4-trifluoro-3-oxobutanoate as a brown oil (900 mg, crude) that was used as such for the next step without any further purification. MS: 273.0 (M-H)^+^. (ii) Et_3_N (2.0 mL, 14.5 mmol) was added to a solution of ethyl 2-benzyl-4,4,4-trifluoro-3-oxobutanoate (800 mg, 2.9 mmol) and picolinimidamide hydrochloride (460 mg, 2.9 mmol) in EtOH (10 mL) at RT. The reaction mixture was then heated at 100°C and monitored by TLC analysis (50% EtOAc-hexane) until completion. The reaction mixture was then poured into ice water (20 g) and extracted with EtOAc (3 × 30 mL). The combined organic layer was dried over anhydrous Na_2_SO_4_ and concentrated under reduced pressure. The crude was purified by flash chromatography on silica gel (100–200 mesh) using 8% EtOAc-hexane as eluent to afford **52** as a white solid (100 mg, 11.0%). M.P. 173–174°C. ^1^H NMR (400 MHz, DMSO-d_6_): δ 12.99 (s, 1H), 8.77 (d, *J* = 4.2 Hz, 1H), 8.31 (d, *J* = 7.9 Hz, 1H), 8.06–8.10 (m, 1H), 7.67–7.70 (m, 1H), 7.25–7.29 (m, 2H), 7.17–7.21 (m, 3H), 4.01 (s, 2H); ^13^C NMR (100 MHz, DMSO-d_6_): δ 162.1, 153.8, 149.2, 147.8, 146.9 (d, *J* = 35 Hz), 138.2, 138.1, 128.3, 127.9, 127.1, 126.9, 126.2, 122.7, 121.6 (d, *J* = 274 Hz), 29.9. LCMS (ESI) m/z calculated for [C_17_H_12_F_3_N_3_O + H]^+^, 332.10; found 332.95.

### Synthesis of 5-(4-Fluorophenyl)-2-(Pyridin-2-yl)-6-(Trifluoromethyl)Pyrimidin-4(3H)-One (53, 0504061)

(i) Synthesis of ethyl 4,4,4-trifluoro-2-(4-fluorophenyl)-3-oxobutanoate: 2-(4-Fluorophenyl)acetyl chloride (1.0 g, 5.8 mmol) was taken in DCM (10 mL) in a 100-mL round-bottom flask under N_2_. TFAA (1.2 mL, 8.7 mmol) and pyridine (1.0 mL, 11.6 mmol) were sequentially added to it. The reaction mixture was stirred at RT for 3 h. EtOH (4.0 mL) was added to the reaction mixture and kept it stirring for another 12 h. The reaction mixture was concentrated under reduced pressure and triturated with hexane. This afforded ethyl 4,4,4-trifluoro-2-(4-fluorophenyl)-3-oxobutanoate as a brown oil (1.0 g, crude) that was used as such for the next step without any further purification. MS: 276.8 (M-H^+^).(ii) Et_3_N (5.2 mL, 36.0 mmol) was added to a solution of ethyl 4,4,4-trifluoro-2-(4-fluorophenyl)-3-oxobutanoate (2.0 g, 7.2 mmol) and picolinimidamide hydrochloride **3** (1.1 g, 7.2 mmol) in EtOH (20 mL) at RT. The reaction mixture was then heated at 100°C for 12 h and monitored by TLC analysis (50% EtOAc-hexane). The reaction mixture was then poured into ice water (20 g) and extracted with EtOAc (3 × 30 mL). The combined organic layer was dried over anhydrous Na_2_SO_4_ and concentrated under reduced pressure. The crude was purified by flash chromatography on silica gel (100–200 mesh) using 25% EtOAc-hexane as eluent to afford **53** as a white solid (115 mg, 5.0%). M.P. 217–220°C. ^1^H NMR (400 MHz, DMSO-d_6_): δ 13.08 (s, 1H), 8.79 (d, *J* = 4.2 Hz, 1H), 8.35 (d, *J* = 7.9 Hz, 1H), 8.08–8.12 (m, 1H), 7.69–7.72 (m, 1H), 7.34–7.38 (m, 2H), 7.27–7.31 (m, 2H); ^13^C NMR (100 MHz, DMSO-d_6_): δ 162.1 (d, *J* = 244 Hz), 161.5, 154.7, 149.4, 147.8, 146.3 (q, *J* = 32 Hz), 138.2, 131.7 (d, *J* = 8.0 Hz), 127.4, 127.2, 122.8, 121.2 (q, *J* = 275 Hz), 114.9 (d, *J* = 21 Hz). LCMS (ESI) m/z calculated for [C_16_H_9_F_4_N_3_O + H]^+^, 336.08; found 335.91.

### Synthesis of 5-Phenyl-2-(Pyridin-2-yl)-6-(Trifluoromethyl)Pyrimidin-4(3H)-One (54, 0504060)

(i) Synthesis of ethyl 4,4,4-trifluoro-3-oxo-2-phenylbutanoate (93): 2-Phenylacetyl chloride (2.0 g, 12.9 mmol) was taken in DCM (20 mL) in a 250-mL round-bottom flask under N_2_. TFAA (2.7 mL, 19.4 mmol) and pyridine (2.1 mL, 25.8 mmol) were sequentially added to it. The reaction mixture was stirred at RT for 3 h. EtOH (4.0 mL) was added to the reaction mixture and kept it stirring for another 12 h. The reaction mixture was concentrated under reduced pressure and triturated with hexane. This afforded ethyl 4,4,4-trifluoro-3-oxo-2-phenylbutanoate as a brown oil (2.0 g, crude) that was used as such for the next step without any further purification. MS: 258.8 (M-H^+^). (ii) Et_3_N (5.5 mL, 38.5 mmol) was added to a solution of ethyl 4,4,4-trifluoro-3-oxo-2-phenylbutanoate (2.0 g, 7.7 mmol) and picolinimidamide hydrochloride (1.2 g, 7.7 mmol) in EtOH (10 mL) at RT. The reaction mixture was then heated at 100°C for 12 h and monitored by TLC analysis (50% EtOAc-hexane). The reaction mixture was then poured into ice water (20 g) and extracted with EtOAc (3 × 30 mL). The combined organic layer was dried over anhydrous Na_2_SO_4_ and concentrated under reduced pressure. The crude was purified by flash chromatography on silica gel (100–200 mesh) using 24% EtOAc-hexane as eluent to afford **54** as a white solid (70 mg, 3.0%). M.P. 258–259°C. ^1^H NMR (400 MHz, DMSO-d_6_): δ 13.04 (s, 1H), 8.79 (d, *J* = 4.2 Hz, 1H), 8.35 (d, *J* = 7.8 Hz, 1H), 8.08–8.12 (m, 1H), 7.69–7.72 (m, 1H), 7.41–7.48 (m, 3H), 7.29–7.31 (m, 2H); ^13^C NMR (100 MHz, DMSO-d_6_): δ 161.5, 154.6, 149.4, 147.8, 146.5 (d, *J* = 32 Hz), 138.2, 131.2, 129.4, 128.4, 127.8, 127.2, 122.8, 121.3 (q, *J* = 274 Hz). LCMS (ESI) m/z calculated for [C_16_H_10_F_3_N_3_O + H]^+^, 318.09; found 317.94.

### Synthesis of 5-Ethyl-2-(Pyridin-2-yl)-6-(Trifluoromethyl)Pyrimidin-4(3H)-One (55, 0497747)

(i) Synthesis of ethyl 2-ethyl-4,4,4-trifluoro-3-oxobutanoate: Butyryl chloride (5 g, 47.2 mmol) was taken in DCM (25 mL) in a 250-mL round-bottom flask under N_2_. TFAA (9.9 mL, 70.7 mmol) and pyridine (7.6 mL, 94.4 mmol) were sequentially added to it. The reaction mixture was stirred at RT for 3 h. EtOH (8 mL) was added to the reaction mixture and kept it stirring for another 12 h. The reaction mixture was concentrated under reduced pressure and triturated with hexane. This afforded ethyl 2-ethyl-4,4,4-trifluoro-3-oxobutanoate as a brown oil (2.0 g, 20%) that was used as such for the next step without any further purification. MS m/z 235.0 [M^−^+ Na]. (ii) Na_2_CO_3_ (2.5 g, 23.5 mmol) was added to a solution of ethyl 2-ethyl-4,4,4-trifluoro-3-oxobutanoate (1.0 g, 4.7 mmol) and picolinimidamide hydrochloride (750 mg, 4.7 mmol) in THF (15 mL) at RT. The reaction mixture was then heated at 70°C for 16 h and monitored by TLC analysis (50% EtOAc-hexane). The reaction mixture was then poured into ice water (20 g) and extracted with EtOAc (2 × 30 mL). The combined organic layer was dried over anhydrous Na_2_SO_4_ and concentrated under reduced pressure. The crude was purified by flash chromatography on silica gel (100–200 mesh) 8% EtOAc-hexane as eluent to afford **55** as a white solid (65 mg, 0.05%). M.P. 122–124°C. ^1^H NMR (400 MHz, DMSO-d_6_): δ 12.85 (s, 1H), 8.76 (d, *J* = 4.6 Hz, 1H), 8.28 (d, *J* = 7.9 Hz, 1H), 8.07 (t, *J* = 7.8 Hz, 1H), 7.65–7.69 (m, 1H), 2.61 (q, *J* = 6.0 Hz, 2H), 1.11 (t, *J* = 7.3 Hz, 3H); ^13^C NMR (100 MHz, DMSO-d_6_): δ 161.7, 153.0, 149.2, 147.8, 145.7 (q, *J* = 32 Hz), 138.1, 130.4, 126.9, 122.5, 121.8 (q, *J* = 275 Hz), 18.4, 12.9. LCMS (ESI) m/z calculated for [C_12_H_10_F_3_N_3_O + H]^+^, 270.09; found 270.43.

### Synthesis of 5-Methoxy-2-(Pyridin-2-yl)-6-(Trifluoromethyl)Pyrimidin-4(3H)-One (56, 0498336)

(i) Synthesis of ethyl 4,4,4-trifluoro-2-methoxy-3-oxobutanoate: 2-Methoxyacetyl chloride (5 g, 46.1 mmol) was taken in DCM (30 mL) in a 250-mL round-bottom flask under N_2_. TFAA (9.8 mL, 69.1 mmol) and pyridine (7.4 mL, 92.2 mmol) were sequentially added to it. The reaction mixture was stirred at RT for 3 h. EtOH (8 mL) was added to the reaction mixture and kept it stirring for another 12 h. The reaction mixture was concentrated under reduced pressure. It was diluted with water and extracted with EtOAC (3 × 30 mL). The combined extract was washed with 10% HCl (3 × 20 mL) followed by water (3 × 20 mL). The combined organic layer was dried over anhydrous Na_2_SO_4_ and concentrated under reduced pressure. This afforded ethyl 4,4,4-trifluoro-2-methoxy-3-oxobutanoate as a brown oil (3.5 g, 35%) that was used as such for the next step without any further purification. (ii) Et_3_N (7.0 mL, 48.9 mmol) was added to a solution of ethyl 4,4,4-trifluoro-2-methoxy-3-oxobutanoate (3.5 g, 16.3 mmol) and picolinimidamide hydrochloride (2.5 g, 16.3 mmol) in EtOH (20 mL) at RT. The reaction mixture was then heated at 100°C and monitored by TLC analysis (50% EtOAc-hexane) until completion. The reaction mixture was then poured into ice water (20 g) and extracted with EtOAc (3 × 30 mL). The combined organic layer was dried over anhydrous Na_2_SO_4_ and concentrated under reduced pressure. The crude was purified by flash chromatography on silica gel (100–200 mesh) using 20% EtOAc-hexane as eluent to afford **56** as a white solid (35 mg, 0.8%). M.P. 175–176°C. ^1^H NMR (400 MHz, DMSO-d_6_): δ 13.04 (s, 1H), 8.74 (d, *J* = 4.2 Hz, 1H), 8.24 (d, *J* = 7.8 Hz, 1H), 8.05 (t, *J* = 7.3 Hz, 1H), 7.63–7.65 (m, 1H), 3.99 (s, 3H); ^13^C NMR (100 MHz, DMSO-d_6_): δ 158.4, 149.6, 149.1, 147.8, 145.9, 138.3, 137.6 (q, *J* = 33 Hz), 126.6, 122.3, 121.3 (q, *J* = 274 Hz), 60.2. LCMS (ESI) m/z calculated for [C_11_H_8_F_3_N_3_O_2_ + H]^+^, 272.06; found 272.30.

### Synthesis of 5-Cyclopentyl-2-(Pyridin-2-yl)-6-(Trifluoromethyl)Pyrimidin-4(3H)-One (57, 0498342)

(i) Synthesis of ethyl 2-cyclopentyl-4,4,4-trifluoro-3-oxobutanoate: 2-Cyclopentylacetyl chloride (3.0 g, 20.5 mmol) was taken in DCM (20 mL) in a 250-mL round-bottom flask under N_2_. TFAA (4.3 mL, 30.7 mmol) and pyridine (3.3 mL, 41.0 mmol) were sequentially added to it. The reaction mixture was stirred at RT for 3 h. EtOH (5 mL) was added to the reaction mixture and kept it stirring for another 12 h. The reaction mixture was concentrated under reduced pressure and triturated with hexane. This afforded ethyl 2-cyclopentyl-4,4,4-trifluoro-3-oxobutanoate as a brown oil (2.0 g, crude) that was used as such for the next step without any further purification. MS: 250.8 (M-H)^+^. (ii) Et_3_N (5.7 mL, 39.5 mmol) was added a solution of ethyl 2-cyclopentyl-4,4,4-trifluoro-3-oxobutanoate (2.0 g, 7.9 mmol) and picolinimidamide hydrochloride **3** (1.2 g, 7.9 mmol) in EtOH (20 mL) at RT. The reaction mixture was then heated at 110°C and monitored by TLC analysis (50% EtOAc-hexane) until completion. The reaction mixture was then poured into ice water (20 g) and extracted with EtOAc (3 × 30 mL). The combined organic layer was dried over anhydrous Na_2_SO_4_ and concentrated under reduced pressure. The crude was purified by flash chromatography on silica gel (100–200 mesh) using 20% EtOAc-hexane as eluent to afford **57** as a white solid (200 mg, 8.0%). M.P. 150–151°C. ^1^H NMR (400 MHz, DMSO-d_6_): δ 12.72 (s, 1H), 8.75 (d, *J* = 4.6 Hz, 1H), 8.27 (d, *J* = 7.9 Hz, 1H), 8.06 (t, *J* = 7.8 Hz, 1H), 7.67 (q, *J* = 4.9 Hz, 1H), 3.12–3.19 (m, 1H), 2.03–2.09 (m, 2H), 1.87 (m, 2H), 1.62–1.69 (m, 4H); ^13^C NMR (100 MHz, DMSO-d_6_): δ 160.6, 150.9, 149.2, 147.8, 146.5 (q, *J* = 33 Hz), 138.1, 131.5, 126.9, 122.5, 121.8 (q, *J* = 275 Hz), 37.6, 29.6, 26.6. LCMS (ESI) m/z calculated for [C_15_H_14_F_3_N_3_O + H]^+^, 310.12; found 310.39.

### Synthesis of 5-Cyclohexyl-2-(Pyridin-2-yl)-6-(Trifluoromethyl)Pyrimidin-4(3H)-One (58, 0499233)

(i) Synthesis of ethyl 2-cyclohexyl-4,4,4-trifluoro-3-oxobutanoate: 2-Cyclohexylacetyl chloride (2.0 g, 12.5 mmol) was taken in DCM (20 mL) in a 250-mL round-bottom flask under N_2_. TFAA (2.6 mL, 18.7 mmol) and pyridine (2.0 mL, 25.0 mmol) were sequentially added to it. The reaction mixture was stirred at RT for 3 h. EtOH (4 mL) was added to the reaction mixture and kept it stirring for another 12 h. The reaction mixture was concentrated under reduced pressure and triturated with hexane. This afforded ethyl 2-cyclohexyl-4,4,4-trifluoro-3-oxobutanoate as a brown oil (2.5 g, crude) that was used as such for the next step without any further purification. (ii) Et_3_N (6.8 mL, 47.0 mmol) was added to a solution of ethyl 2-cyclohexyl-4,4,4-trifluoro-3-oxobutanoate (2.5 g, 9.4 mmol) and picolinimidamide (1.1 g, 9.4 mmol) in EtOH (25 mL) at RT. The reaction mixture was then heated at 100°C and monitored by TLC analysis (50% EtOAc-hexane) until completion. The reaction mixture was then poured into ice water (20 g) and extracted with EtOAc (3 × 30 mL). The combined organic layer was dried over anhydrous Na_2_SO_4_ and concentrated under reduced pressure. The crude was purified by flash chromatography on silica gel (100–200 mesh) using 12% EtOAc-hexane as eluent to afford **58** as a white solid (125 mg, 4.0%). M.P. 220–221°C. ^1^H NMR (400 MHz, CDCl_3_): δ 10.79 (s, 1H), 8.66 (d, *J* = 4.0 Hz, 1H), 8.45 (d, *J* = 8.0 Hz, 1H), 7.91 (t, *J* = 8.0 Hz, 1H), 7.50 (q, *J* = 7.3 Hz, 1H), 2.83–2.89 (m, 1H), 2.26–2.32 (m, 2H), 1.82–1.85 (m, 2H), 1.70 (m, 1H), 1.56 (m, 2H), 1.28–1.42 (m, 3H); ^13^C NMR (100 MHz, CDCl_3_): δ 161.2, 151.4, 149.1, 148.2 (q, *J* = 32 Hz), 147.2, 137.9, 134.4, 126.9, 122.4, 121.9 (q, *J* = 278 Hz), 39.1, 29.5, 26.6, 25.7. LCMS (ESI) m/z calculated for [C_16_H_16_F_3_N_3_O + H]^+^, 324.13; found 324.66.

### Bacterial Culture

*M. tuberculosis* H37Rv was grown on Middlebrook 7H10 agar (Becton Dickinson) supplemented with 10% vol/vol oleic acid, albumin, dextrose, and catalase (OADC) (Becton Dickinson) or Middlebrook 7H9 with (Becton Dickinson) OADC and 0.05% wt/vol Tween 80 (7H9-OADC-Tw). *M. tuberculosis* H37Rv constitutively expressing a red fluorescent protein (*Ds*Red) (Carroll et al., 2018) was grown with 50 μg/mL hygromycin B. *Staphylococcus aureus* RN4220 and *Escherichia coli* DH5α were grown on LB agar. *Mycobacterium smegmatis* mc^2^155 was grown on 7H10 with 10% OADC. *Pseudomonas aeruginosa* BAA 47 was grown on tryptic soy agar, *Saccharomyces cerevisiae* Y187 was grown on YPD agar, and *Bacillus subtilis* Marburg was grown on nutrient agar.

### Minimum Inhibitory Concentration

The compound concentration required to inhibit growth of *M. tuberculosis* was determined by testing compounds as 2-fold serial dilutions, typically starting at 20 μM. Growth inhibition was measured after 5 days using a fluorescent strain of *M. tuberculosis* in 7H9-OADC-Tw, according to previously published methods (Ollinger et al., 2013, 2018). Each 96-well plate contained positive and negative controls, as well as a standard curve for rifampicin.

### Cytotoxicity

The compound concentration that resulted in 50% growth inhibition of HepG2 human liver cells (ATCC) was determined by testing compounds as 3-fold serial dilutions, typically starting at 100 μM. Cytotoxicity was measured after 72 h of compound exposure at 37°C and 5% CO_2_ using CellTiter-Glo (Promega). Each 384-well plate contained positive and negative controls, as well as a standard curve for staurosporine.

### Spectrum

Minimum inhibitory concentration to inhibit growth of 99% (MIC_99_) on solid medium was determined using the agar serial dilution method. Late log phase cultures were plated at ~10^5^ colony-forming units (CFU)/mL onto solid medium containing 2-fold serial dilutions of compounds. The MIC_99_ is defined as the lowest concentration with ≤1% growth.

### Kill Kinetics

*M. tuberculosis* was cultured to late log phase. Compound was added (final concentration 2% DMSO), and cultures were incubated standing at 37°C. Viable bacteria were determined by serial dilutions and plating on Middlebrook 7H10 plus 10% vol/vol OADC. CFUs were counted after 3–4 weeks. The untreated control was 2% DMSO.

## Results and Discussion

### Chemistry

**Figure 1:**
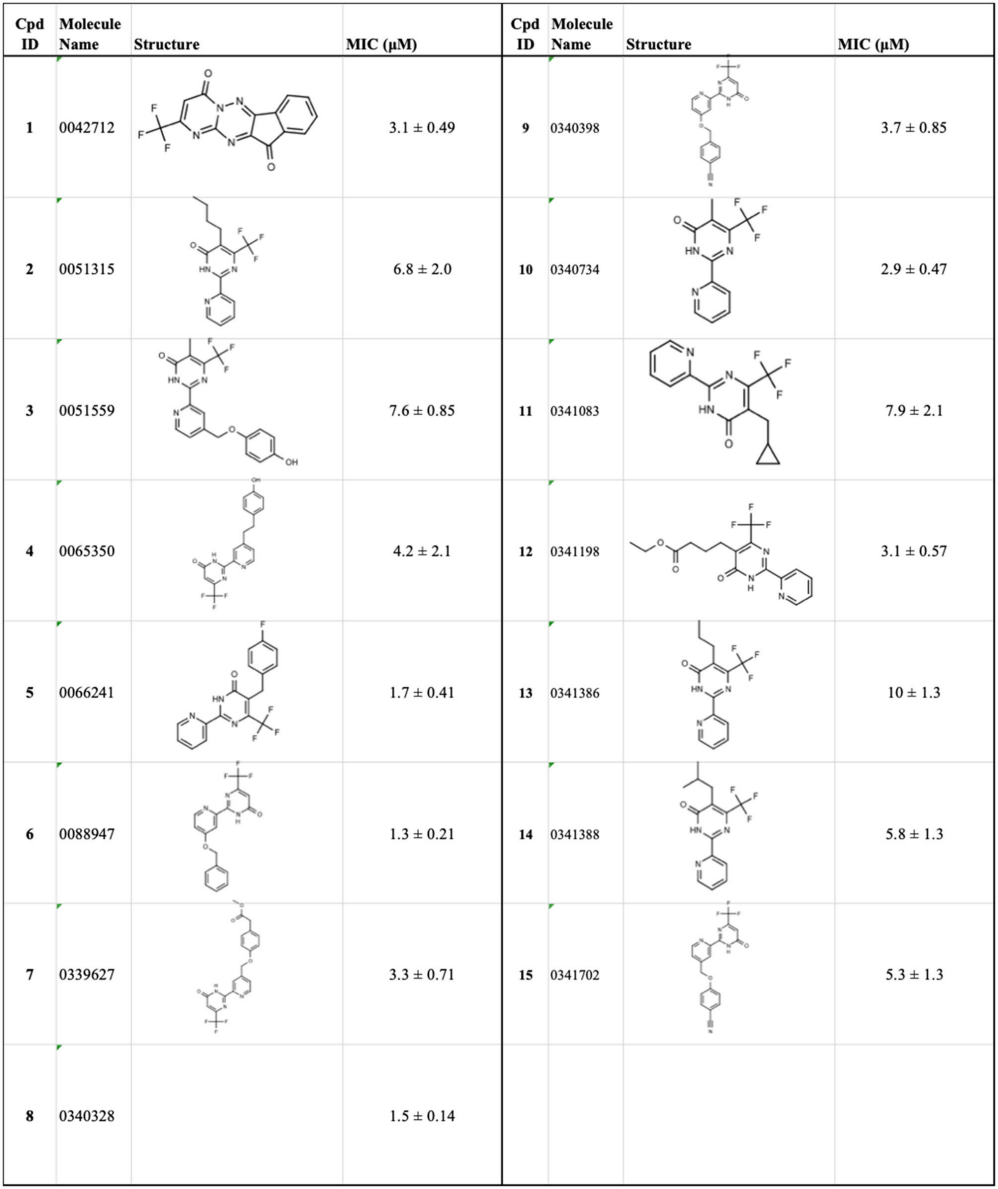
Primary hits. MTB IC_90_ is the concentration required to inhibit *M. tuberculosis* growth by 90%. Data are the average of a minimum of two independent experiments ± standard deviation.

We developed and conducted a whole-cell screen that identified the trifluoromethyl pyrimidinone series as having good growth inhibitory activity against virulent *M. tuberculosis*. We identified 15 compounds with activity in the primary screen. We determined the MIC for each. The primary hits are shown in Figure 1 with MICs. Among the initial set of 15 analogs, several compounds displayed good activity with an IC_90_ of <5.0 (Figure 1). In general, the series had low molecular weight (<500), relatively low hydrophobicity (clogP < 5) and complied with Lipinski's Rule of 5, making this an attractive series for assessment. The ease of synthesis was another attractive feature.

Based on the attractive properties of this series, we expanded our studies to investigate the SAR of the series. Our objective was to explore the limits of the chemical space making structurally diverse molecules to determine if the series had dynamic SAR (>50-fold changes in MIC) and/or if we could improve the anti-tubercular potency (to <1 μM) and reduce cytotoxicity (selectivity index >10). We synthesized a series of substituted pyrimidinone to establish SAR. A small number of publications describe the synthesis of substituted trifluoromethyl pyrimidinones (Fruit and Besson, 2018). We used a procedure similar to those of Tice and Bryman to obtain substituted trifluoromethyl pyrimidinones (Tice and Bryman, 2001); we introduced trifluoromethyl substituents by the condensation of a variety of amidines with substituted ethyl 4,4,4-trifluoro-3-oxobutanoate.

**Scheme 1:**
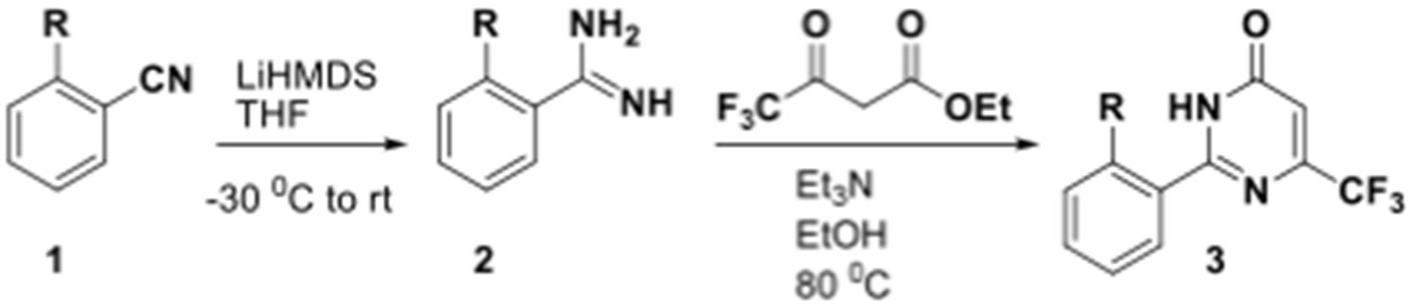
General Scheme for the synthesis of trifluoromethyl pyrimidinone.

We used amidine as the starting material for the synthesis of trifluoromethyl pyrimidinones. Treatment of the substituted aryl or pyridine nitrile, such a picolinonitrile, with lithium *bis*(trimethylsilyl)amide in tetrahydrofuran at −30°C and warming the reaction mixture to an ambient temperature afforded the amidine in moderate to high yield. Reaction of the amidine with ethyl 4,4,4-trifluoro-3-oxobutanoate in the presence of a base in ethanol under refluxing conditions furnished the trifluoromethyl pyrimidinone compound **16** with a high yield (Scheme 1). Following this synthetic pathway, all compounds were prepared in two or three steps from commercially available and inexpensive starting materials.

### Biological Testing

We tested all compounds for activity against *M. tuberculosis* in aerobic culture, as well as for cytotoxicity using the HepG2 cell line.

We first looked at substitutions on the 2-position of the pyrimidinone. The initial SAR is summarized in Table 1. In this set, the most attractive molecule was compound **16**, which had good activity (IC_90_ = 4.9) and a lack of cytotoxicity (IC_50_ > 100), as well as good physicochemical properties with clogP of 0.63 and topological polar surface area of 53.8. It was interesting that this compound had good activity, even though it had a low clogP, as the activity of molecules against *M*. tuberculosis is often limited by their ability to penetrate the waxy cell wall (Machado et al., 2018). Compounds **7** and **8** with substitution of the 2-pyridine rings showed slightly improved potency (IC_90_ = 3.3 and 1.5 μM, respectively), as compared to compound **16**. Inclusion of a polar hydroxyl group at the 3-position of the pyridine ring was tolerated (compound **17**, IC_90_ = 3.3 μM). However, all of these substitutions increased cytotoxicity with IC_50_ < 20 μM against HepG2 cells. Addition of a benzyloxy at the 4-position of pyridyl ring retained potency (compound **6**, IC_90_ = 1.3 μM) but did not improve cytotoxicity (IC_50_ = 2.5 μM). Incorporating a nitrile on the benzyloxy group (compound **9**) or the reverse benzyloxy group (**15**) similarly retained activity (IC_90_ = 3.7 and 5.3 μM, respectively), but with no improvement on cytotoxicity (IC_50_ = 6.1 and 7.4 μM, respectively). Replacing the oxygen atom linker on the pyridine ring with a carbon chain such as 2-(4-(4-hydroxyphenethyl)pyridin-2-yl in compound **4** decreased potency (IC_90_ = 7.8 μM). However, replacing the 2-pyridyl ring with a 2-pyrimidine ring in **18** abolished activity and cytotoxicity.

**Table 1:** Activity of compounds with substitutions at the 2-position of the pyrimidinone.

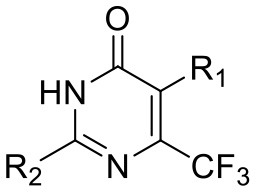					
**Compound**	**Molecule identifier**	**R**_**1**_	**R**_**2**_	**MTB**	**HepG2**
				${\text{IC}}_{90}^{\text{a}}$ **(μM)**	${\text{IC}}_{50}^{\text{b}}$ **(μM)**
6^*^	0088947	H	2-(4-(benzyloxy)pyridin-2-yl)	1.3 ± 0.21 (*n =* 2)	2.5 ± 0.78 (*n =* 2)
7^*^	0339627	H	5-CH_2_O-2′-CH_2_CO_2_CH_3_-Ph-2-Pyridyl	3.3 ± 0.71 (*n =* 2)	7.1 ± 0.14 (*n =* 2)
8^*^	0340328	H	5-CH_2_O-2′-CO_2_CH_3_-Ph-2-Pyridyl	1.5 ± 0.14 (*n =* 2)	5.4 ± 1.4 (*n =* 2)
9^*^	0340398	H	4′-CN-5-BnO-2-Pyridyl	3.7 ± 0.85 (*n =* 2)	6.1 ± 2.1 (*n =* 2)
15^*^	0341702	H	5-CH_2_O-4′-CN-Ph-2-Pyridyl	5.3 ± 1.3 (*n =* 2)	7.4 ± 1.6 (*n =* 2)
**16**	0496338	H	2-Pyridyl	4.9 ± 0.79 (*n =* 6)	>100 (*n =* 3)
**17**	0504065	H	3-Hydroxypyridin-2-yl	3.3 ± 1.1 (*n =* 2)	16 ± 7.6 (*n =* 3)
**18**	0504070	H	2-(Pyrimidin-2-yl)	>20 (*n =* 2)	>100 (*n =* 2)
**19**	0499009	H	3-Methoxypyridin-2-yl	>20 (*n =* 2)	>100 (*n =* 2)
**20**	0497740	H	Pyridin-3-yl	>20 (*n =* 2)	>100 (*n =* 3)
**21**	0498345	H	3-Chloropyridin-2-yl	>20 (*n =* 2)	>100 (*n =* 2)
**22**	0497401	Me	3-pyridyl	>20 (*n =* 2)	>100 (*n =* 3)
**23**	0497305	Me	4-pyridyl	>20 (*n =* 4)	>100 (*n =* 2)
**24**	0143990	H	Ph	>20 (*n =* 2)	>100 (*n =* 2)
**25**	0195120	H	2-(Trifluoromethoxy)phenyl	>20 (*n =* 2)	>100 (*n =* 3)
**26**	0499232	H	3-Fluoropyridin-2-yl	>20 (*n =* 2)	>100 (*n =* 4)
**27**	0504094	H	Cyclohexyl	>20 (*n =* 2)	90 ± 17 (*n =* 3)
**28**	0497802	H	2-Fluorophenyl	>20 (*n =* 2)	>100 (*n =* 2)
**29**	0504068	H	2,6-Dimethylphenyl	>20 (*n =* 2)	>100 (*n =* 2)
**30**	0499224	H	2-*o*-Tolyl-	>20 (*n =* 2)	>100 (*n =* 2)
**31**	0498338	H	2-Hydroxyphenyl	>20 (*n =* 2)	79 ± 7.8 (*n =* 2)

*^a^MTB IC_90_ is the concentration required to inhibit M. tuberculosis growth by 90%. ^b^HepG2 IC_50_ is the concentration required to inhibit growth of HepG2 cells by 50%. Data are the average of a minimum of two independent experiments ± standard deviation*.

* *Compounds identified in the primary screen. For comparison, the MIC of rifampicin in this assay is 8 ± 0.2 nM (n = 129), for isoniazid is 0.6 ± 0.2 μM (n = 21), and for ethambutol is 3.3 ± 0.4 μM (n = 12)*.

In order to rationalize SAR and obtain additional information on cytotoxicity liability, we designed a small set of library analogs around compound **16**. Analogs with phenyl rings were prepared using cyclocondensation conditions using a variety of amidines as described in Scheme 1. Interestingly, moving the nitrogen around the pyridyl ring and substitution around the pyridyl ring led to loss of anti-tubercular activity (IC_90_ >20 μM) as shown in **19–23**. We replaced the pyridyl ring with a substituted phenyl ring in **24–31**. As we had seen a trend toward increased potency with a 2-pyridine ring and any substitution at the C-4 position, we used this strategy to increase potency. However, we did not see improved potency with either unsubstituted or substituted phenyl groups (**24–31)**, where all compounds were inactive (IC_90_ > 20 μM).

We next explored substitution on the pyrimidinone ring at C-5 using a methyl moiety as R1 (**32–46**, Table 2) and varying substitution at C-2 as R2 (**47–50**, Table 3). Several compounds were synthesized following the same cyclocondensation conditions with a variety of amidines and substituted ethyl 4,4,4-trifluoro-3-oxobutanoate as depicted in Scheme 1 to obtain **32–46**. Compound **32** without substitution at C-3 on the pyrimidinone core had no antibacterial activity. Similarly, loss of potency was observed for compounds **34–46** (Table 2) with trifluoromethyl and methyl groups at C-5 and C-6, respectively, and different substitutions at C-3. The methyl group did not appear to affect potency, as the matched pair of compounds with **3** and **4** had similar potency and cytotoxicity (within 2-fold). Similar to the SAR observed for the 2-pyridine with hydrogen as R1, we found a trend toward increased potency with 2-pyridine analogs; compounds **10** and **34** had similar potency (IC_90_ = 2.9 and 2.5 μM, respectively) as **16** and **17**. However, both **10** and **33** were cytotoxic with IC_50_ of 5.4 and 3.2 μM, respectively. As with compound **18**, compound **43** containing a pyrimidine group showed no cytotoxicity, but no antibacterial activity either.

**Table 2:** Activity of compounds with substitutions at the 2- and 5-positions of the pyrimidinone.

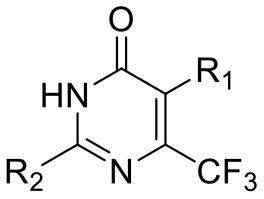					
**Compound**	**Molecule identifier**	**R**_**1**_	**R**_**2**_	**MTB**	**HepG2**
				${\text{IC}}_{90}^{\text{a}}$ **(μM)**	${\text{IC}}_{50}^{\text{b}}$ **(μM)**
3^*^	0051559	Me	5-CH2O-4′-OH-Ph-2-pyridyl	7.6 ± 0.85 (*n =* 2)	7.4 ± 0.28 (*n =* 2)
10^*^	0340734	Me	2-Pyridyl	2.9 ± 0.47 (*n =* 5)	5.4 ± 3.1 (*n =* 8)
**32**	0496404	Me	H	>20 (*n =* 2)	>100 (*n =* 2)
**33**	0504069	Me	3-Hydroxypyridin-2-yl	2.5 ± 1.1 (*n =* 3)	3.2 ± 1.6 (*n =* 2)
**34**	0497565	Me	2-Methoxyphenyl	>20 (*n =* 2)	95 ± 9.5 (*n =* 4)
**35**	0496408	Me	Ph	>20 (*n =* 2)	>100 (*n =* 3)
**36**	0496405	Me	2-Fluorophenyl	>20 (*n =* 2)	>100 (*n =* 3)
**37**	0497560	Me	2-Cholorphenyl	>20 (*n =* 2)	86 ± 24 (*n =* 3)
**38**	0498337	Me	3-Chloropyridin-2-yl	>20 (*n =* 2)	>100 (*n =* 2)
**39**	0499223	Me	3-Methoxypyridin-2-yl	>20 (*n =* 2)	>100 (*n =* 2)
**40**	0504095	Me	Cyclohexyl	>20 (*n =* 2)	>100 ± (*n =* 3)
**41**	0504071	Me	2-(Pyrimidin-2-yl)	>20 (*n =* 2)	>100 ± (*n =* 2)
**42**	0498344	Me	6-(Trifluoromethyl)	>20 (*n =* 2)	>100 (*n =* 2)
**43**	0504067	Me	2,6-dimethylphenyl	>20 (*n =* 2)	>100 (*n =* 2)
**44**	0504066	Me	(2-(Trifluoromethoxy)phenyl)	>20 (*n =* 2)	>100 (*n =* 2)
**45**	0504056	Me	3-Fluoropyridin-2-yl	>20 (*n =* 2)	>100 (*n =* 4)
**46**	0499007	Me	2-Hydroxyphenyl	>20 (*n =* 2)	44 ± 7.8 (*n =* 2)

*^a^MTB IC_90_ is the concentration required to inhibit M. tuberculosis growth by 90%. ^b^HepG2 IC_50_ is the concentration required to inhibit growth of HepG2 cells by 50%. Data are the average of a minimum of two independent experiments ± standard deviation*.

* *Compounds identified in the primary screen*.

**Table 3:** Activity of compounds with 2-(pyridin-2-yl)pyrimidin-4(3H)-ones.

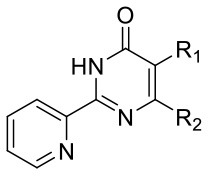					
**Compound**	**Molecule identifier**	**R**_**1**_	**R**_**2**_	**MTB**	**HepG2**
				${\text{IC}}_{90}^{\text{a}}$ **(μM)**	${\text{IC}}_{50}^{\text{b}}$ **(μM)**
**47**	0497408	Me	Bn	5.7 ± 0.14 (*n =* 2)	9.8 ± 5.7 (*n =* 4)
**48**	0496303	Me	Ph	9.4 ± 0.67 (*n =* 5)	9.7 ± 5.5 (*n =* 3)
**49**	0496407	Me	Me	>20 (*n =* 2)	88 ± 6.9 (*n =* 3)
**50**	0496336	Me	H	>20 (*n =* 3)	50 ± 13 (*n =* 3)

*^a^MTB IC_90_ is the concentration required to inhibit M. tuberculosis growth by 90%. ^b^HepG2 IC_50_ is the concentration required to inhibit growth of HepG2 cells by 50%. Data are the average of a minimum of two independent experiments ± standard deviation*.

The SAR clearly shows a preference for a 2-pyridine substitution on the pyrimidinone core at C-2 position and the tolerance for a methyl group at the C-5 position of the pyrimidinone moiety. We used this information to design compounds to investigate the role of trifluoromethyl group at the C-6 position. We prepared several compounds (**47–50**) containing a 2-pyridine at C-2 and various group at C-6 position of the pyrimidinone ring (Table 3). Compounds were synthesized using the same scheme as for compound **16**. Compound **47** containing benzyl group at C-6 position was slightly less active, then the matched pair compound with the trifluoromethyl group (**10)**, with an IC_90_ = 5.7 μM compared to IC_90_ = 2.9 μM. Similarly, shortening the linker of the benzyl group to phenyl afforded **48**, which was 3-fold less active (IC_90_ = 9.4 μM). Neither modification improved cytotoxicity (IC_50_ of 9.8 and 9.7 μM, respectively). Another set of matched pairs demonstrated the importance of the trifluoromethyl at C-5 vs. an electron-rich methyl group at the same position (compounds **49** and **51)**: compound **49** did not have any anti-tubercular activity (IC_90_ > 20 μM) compared to compound **51** with an IC_90_ = 2.9 μM. Similarly, compound **50** with a hydrogen atom replacing the trifluoromethyl at the C-5 position of the pyrimidinone ring was inactive (IC_90_ > 20 μM).

Based on the initial SAR interrogation, we determined that the 2-pyridine ring at C-2 position and the trifluoromethyl group at C-4 are critical for activity. Therefore, we focused on the synthesis of substituted pyrimidinone keeping pyridine at C-2 and trifluoromethyl moiety at C-4 and varying the R_1_ group. Following the same synthetic strategies as outlined in Scheme 1, we synthesized a small set of compounds with substitution at C-5 of the pyrimidinone ring (**51–58**). The potency of **51** with a simple methyl group at the C-4 position was comparable to **16** (IC_90_ of 2.9 and 4.9 μM, respectively) (Table 4). Unfortunately, **51** picked up cytotoxicity against HepG2 cell with an IC_50_ of 7.3 μM, whereas **16** lacked cytotoxicity. Addition of a benzyl group at the C-4 position of the pyrimidinone (**52)** did not affect either potency or reduce cytotoxicity. Similarly, analog **5** with a 4-fluorobenzyl group had good potency (IC_90_ = 1.7 μM) but was also cytotoxic (IC_50_ = 3.2 μM). Removal of the methylene linker as in **53** and **54** resulted in a small loss of potency (IC_90_ = 3.8 μM) and a small reduction in cytotoxicity (IC_50_ = 11 μM). We tested additional compounds with a lipophilic R1 group on the pyrimidinone ring to eliminate cytotoxicity. Unfortunately, lipophilicity (**2, 11, 13, 14, 55, 57–58**) or polarity like methoxy and ester groups (**12** and **56**) did not improve potency or eliminate cytotoxicity (Table 4). A small change of substitution pattern from simple methyl (51) to ethyl (55) group at C-4 of the pyrimidinone ring showed comparable anti-tubercular activity (IC_90_ of 2.9 and 3.7 μM, respectively) (Table 4) but retained cytotoxicity. Further addition of the lipophilic groups cyclopropylmethyl (**57**), cyclopentyl (**11**), and cyclohexyl (**58**) did not improve potency or cytotoxicity.

**Table 4:** Activity of compounds with 5/6-position substitutions of the 2-(pyridin-2-yl)pyrimidin-4(3H)-one.

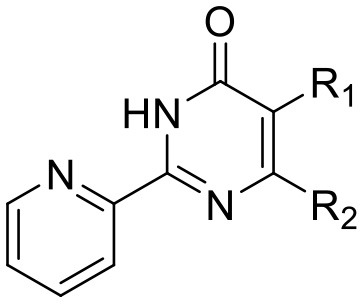					
**Compound**	**Molecule identifier**	**R**_**1**_	**R**_**2**_	**MTB**	**HepG2**
				${\text{IC}}_{90}^{\text{a}}$ **(μM)**	${\text{IC}}_{50}^{\text{b}}$ **(μM)**
2^*^	0051315	Butyl	CF_3_	9.4 ± 3.6 (*n =* 4)	5.3 ± 1.2 (*n =* 2)
5^*^	0066241	4-Fluorobenzyl	CF_3_	1.7 ± 0.41 (*n =* 7)	3.2 ± 1.5 (*n =* 5)
11^*^	0341083	Cyclopentane	CF_3_	7.9 ± 2.1 (*n =* 2)	11 ± 4.7 (*n =* 2)
12^*^	0341198	Ethyl butanoate	CF_3_	3.1 ± 0.57 (*n =* 5)	10 ± 4.3 (*n =* 6)
13^*^	0341386	Propyl	CF_3_	10 ± 1.3 (*n =* 2)	12 ± 1.4 (*n =* 2)
14^*^	0341388	Isobutyl	CF_3_	5.8 ± 1.3 (*n =* 2)	7.5 ± 1.3 (*n =* 2)
**16**	0496338	H	CF_3_	4.9 ± 0.79 (*n =* 6)	>100 (*n =* 3)
**51**	0340734	Me	CF_3_	2.9 ± 0.47 (*n =* 5)	5.7 ± 3.2 (*n =* 8)
**52**	0504059	Bn	CF_3_	2.8 ± 0.78 (*n =* 2)	7.3 ± 4.2 (*n =* 5)
**53**	0504061	4-Fluorophenyl	CF_3_	3.8 ± 1.6 (*n =* 3)	11 ± 6.9 (*n =* 2)
**54**	0504060	Phenyl	CF_3_	4.5 ± 0.14 (*n =* 2)	10 ± 6.4 (*n =* 3)
**55**	0497747	Et	CF_3_	3.7 ± 0.26 (*n =* 5)	6.1 ± 1.3 (*n =* 5)
**56**	0498336	OMe	CF_3_	6.1 ± 0.26 (*n =* 3)	5 ± 0.82 (*n =* 4)
**57**	0498342	Cyclopropylmethyl	CF_3_	10 ± 2.6 (*n =* 2)	5.9 ± 0.57 (*n =* 2)
**58**	0499233	Cyclohexyl	CF_3_	4.1 ± 0.28 (*n =* 2)	2.9 ± 0.28 (*n =* 2)

*^a^MTB IC_90_ is the concentration required to inhibit M. tuberculosis growth by 90%. ^b^HepG2 IC_50_ is the concentration required to inhibit growth of HepG2 cells by 50%. Data are the average of two independent experiments ± standard deviation*.

* *Compounds identified in the primary screen*.

We tested a set of five compounds against a range of bacterial species and yeast (Table 5). Compounds were inactive against the Gram-negative species (*E. coli, P. aeruginosa*, and *K. pneumoniae*), but demonstrated good activity against the Gram-positive species (*S. aureus* and *B. subtilis*), as well as the representative yeast *S. cerevisiae*. Interestingly, compounds were only weakly active against the related non-pathogenic species *M. smegmatis*. This suggests the compounds have broad activity and a common target and/or mode of action against a range of organisms.

**Table 5:** Spectrum of activity.

**Compound**	***M. smegmatis***	***E. coli***	***P. aeruginosa***	***K. pneumoniae***	***S. aureus***	***B. subtilis***	***S. cerevisiae***
**5**	>100	>100	>100	>100	1.6, 1.6	12.5, 6.3	3.1, 3.1
**10**	25	>100	>100	>100	12.5, 12.5	12.5, 12.5	6.3, 12.5
**33**	25	>100	>100	>100	3.1, 3.1	3.1, 6.3	3.1, 3.1
**53**	>100	>100	>100	>100	3.1, 3.1	6.3, 6.3	3.1, 6.3
**55**	100	>100	>100	>100	6.3, 5.0	6.3, 5.0	3.1, 5.0

*MIC_99_s were measured on solid medium and are reported as the minimum concentration required to reduce CFUs by 99%. Data are from two independent runs (where compounds were inactive in both runs, >100 is shown once for clarity)*.

**Figure 2:**
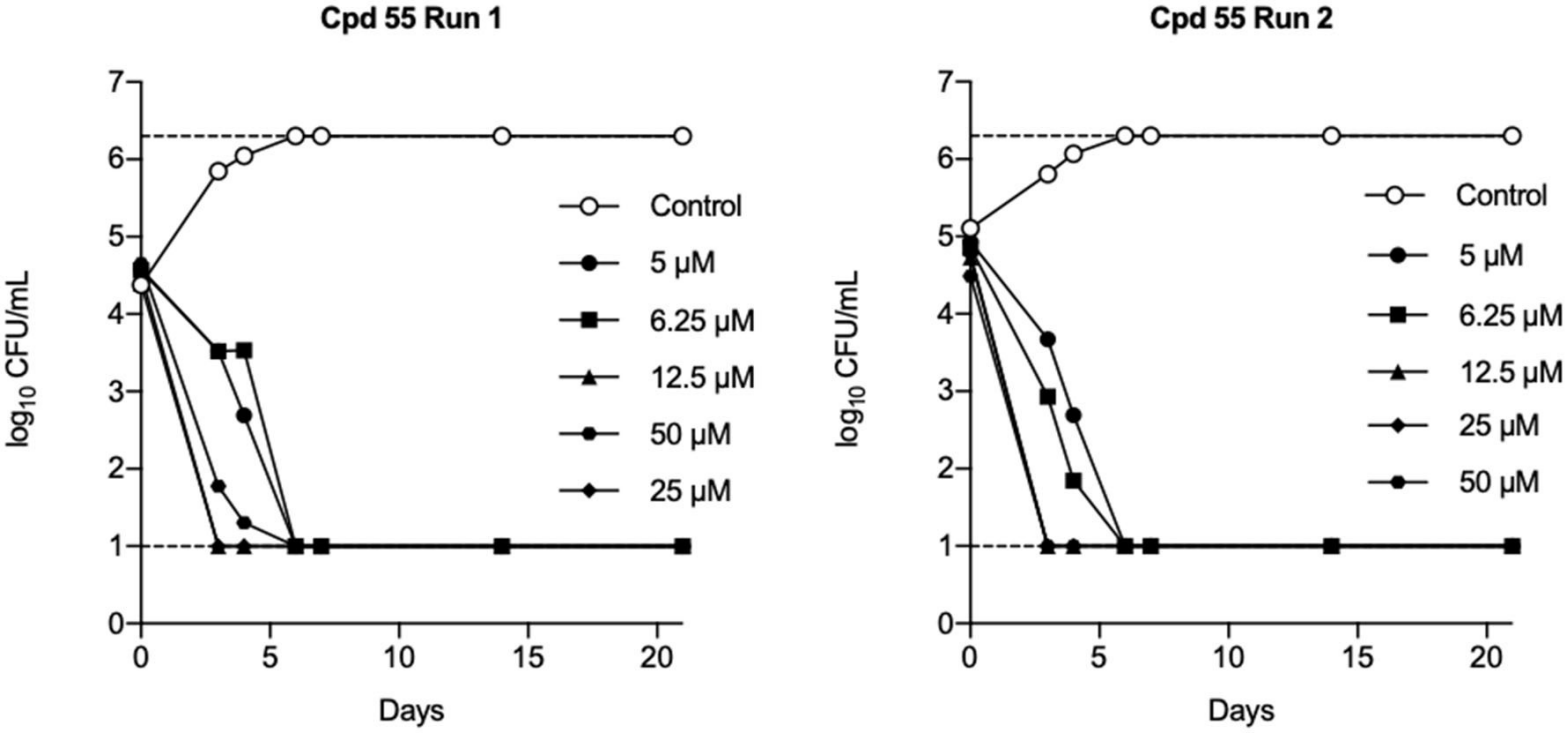
Kill kinetics for a representative compound. *M. tuberculosis* was cultured aerobically and exposed to compound **55**. Viable bacteria were determined by serial dilution and plating onto agar plates for 3 to 4 weeks. Independent experiments are shown. The control was 2% DMSO. The upper and lower limits of detection are indicated.

We selected one representative compound (**55**) and determined kill kinetics against replicating *M. tuberculosis*. We conducted the experiment twice using independent cultures. The compound was rapidly bactericidal leading to ~4 logs reduction in viable bacteria in 7 days (limit of detection) at concentrations as low as 5 μM (Figure 2). Bactericidal activity was concentration dependent with higher kill rates apparent at higher concentrations.

## Conclusion

We identified the trifluoropyrimidinone pharmacophore as active against *M. tuberculosis* in aerobic culture with rapid bactericidal activity and have determined SAR. This series has not been previously tested against *M. tuberculosis*. The series shows a good dynamic range of activity, with the most active being **5** (4-fluorobenzyl), **6** (2-(4-(benzyloxy)pyridin-2-yl), and **8** (methyl 3-((2-(6-oxo-4-(trifluoromethyl)-1,6-dihydropyrimidin-2-yl)pyridin-4-yl) methoxy) benzoate) with IC_90_s of 1.6, 1.3, and 1.5 μM, respectively. The molecules have good calculated properties with low molecular weight, clogP <5, and drug-likeness features of pyrimidinone. Cytotoxicity was an issue with the series, but there is significant scope for further medicinal chemistry optimization to explore SAR and improve in cytotoxicity without any major change in structural features.

## Data Availability Statement

The original contributions presented in the study are included in the article/supplementary material, further inquiries can be directed to the corresponding author/s.

## Author Contributions

EH, JE, JO, MM, TM, PH, and TP contributed to conception and design of the study. CS, OA, JG, LF, DD, MM, AK, and YO designed and conducted the experimental work. TP wrote the first draft of the manuscript. All authors contributed to data analysis, manuscript revision, read, and approved the submitted version.

## Conflict of Interest

EH, TM, and PH were employed by Eli Lilly and Company. PO was employed by company Apollo Drug Discovery Consulting, LLC. The remaining authors declare that the research was conducted in the absence of any commercial or financial relationships that could be construed as a potential conflict of interest.
